# Impact of a shoulder exosuit on range of motion, endurance, and task execution in users with neurological impairments

**DOI:** 10.1017/wtc.2025.10024

**Published:** 2025-08-11

**Authors:** Adrian Esser, Fabian Müller, Julia Manczurowsky, Christopher J. Hasson, Tim Unger, Chris Easthope Awai, Peter Wolf, Robert Riener

**Affiliations:** 1 Sensory Motor Systems Lab, Department of Health Sciences and Technology, ETH Zürich, Zürich, Switzerland; 2 Department of Physical Therapy, Movement and Rehabilitation Science, Northeastern University, Boston, MA, USA; 3 Institute for Experiential Robotics, Northeastern University, Boston, MA, USA; 4 Rehabilitation Engineering Laboratory (RELab), Zürich, Switzerland; 5 Data Analytics and Rehabilitation Technology (DART), Lake Lucerne Institute, Vitznau, Luzern, Switzerland; 6 Spinal Cord Injury Center, Balgrist University Hospital, University of Zurich, Zürich, Switzerland

**Keywords:** activities of daily living, assistance, exosuit, neurorehabilitation, therapy, wearable technology

## Abstract

The Myoshirt, an active exosuit, provides gravity compensation for the shoulders. This study evaluated the impact of the Myoshirt on range of motion (ROM), endurance, and activities of daily living (ADLs) performance through tests involving nine participants with varying levels of arm impairments and diverse pathologies. Optical motion capture was used to quantify ROM of the shoulder and elbow joints during isolated movements and functional tasks. Endurance was quantified through a timed isometric shoulder flexion task, and a battery of ADL tasks was used to measure the perceived support of the exosuit, along with changes in movement quality. Feedback and usability insights were gathered with surveys. The Myoshirt did not significantly improve ROM during isolated movements (shoulder flexion, shoulder abduction, and elbow flexion/extension), but during the reaching phase of a functional drinking task elbow extension increased significantly by 13.5% (t = 7.52, p = .002). Participants could also keep their arms elevated 78.7% longer (t = 1.942, p = .047). Patients also reported less perceived difficulty with ADLs while using the device, and a therapist reported improved execution quality. Participants who self-reported severe impairment levels tended to derive greater benefits compared to those with milder impairments. These findings highlight the potential of the Myoshirt as an assistive device, particularly for individuals with severe impairments, while emphasizing the need for further refinement.

## 1. Introduction

Upper limb (UL) proximal shoulder impairments encompass a wide range of conditions resulting from neurological, musculoskeletal, or other pathological causes. These impairments significantly reduce independence and quality of life (Mazidi et al., 2021). Globally, millions of individuals are affected by UL dysfunction each year. Stroke, for instance, is a leading cause of UL impairments, with 12.2 million new cases annually and 101 million stroke survivors worldwide, leading to an estimated global cost of over US$721 billion (Feigin et al., 2021, 2022). Additionally, spinal cord injury and traumatic brain injury contribute to UL impairments (Guan et al., 2023). Other neurological conditions, like myopathies, muscular dystrophy, sarcopenia, and orthopedic lesions such as brachial plexus injury or torn rotator cuffs, also contribute to UL dysfunction by affecting muscle strength, coordination, and overall mobility.

These wide-ranging effects of UL impairments highlight the challenges faced by individuals in performing daily tasks and maintaining independence. Addressing these challenges has long been a priority in rehabilitation, and with advances in technology, the development of assistive devices has emerged as a promising solution. These devices aim not only to support rehabilitation but also to provide general assistance to individuals with chronic impairments, to enhance the quality of life and functional capacity.

Historically, rehabilitation has utilized methods such as therapist-guided movement with passive mechanisms to alleviate the physical burden of arm movements. These approaches have evolved into more advanced stationary commercial systems like DIEGO (Pavan et al., 2024) and ArmeoSpring (Hocoma, 2024). These systems have shown considerable promise in improving range of motion (ROM), increasing endurance, and facilitating repetitive task execution, demonstrating their value in controlled clinical settings (Fluet and Deutsch, 2011; Mayr et al., 2018; Mehrholz et al., 2018).

Research highlights the impact of gravity support on reducing the effort needed for movements like shoulder abduction, which in turn expands the reachable planar area of the hand (Sukal et al., 2007) and enhances other functional capabilities, such as elbow and finger extension (Chen and Lum, 2016). However, the degree of benefit often correlates with the severity of the impairment, with more pronounced improvements observed in individuals with greater dysfunction (Runnalls et al., 2019). Despite their effectiveness, stationary systems are limited to structured environments and cannot accommodate the dynamic needs of users in daily life, prompting a shift toward portable and wearable solutions. Wearable devices, such as exosuits, mark a significant advancement in assistive technology by prioritizing portability, comfort, and adaptability (Chiaradia et al., 2019). While rigid exoskeletons can fully support the limbs against gravity, soft exosuits provide partial support. Having less support is a trade-off for being able to train exercises or practice functional tasks with a support device that can move with the user and be used both in clinical and home settings. The same system a patient uses in clinic can later be taken home to train functional tasks or be used for daily assistance.

The goal of this study was to evaluate the immediate impact of an UL exosuit on UL mobility and functionality in individuals with chronic UL impairments. The exosuit used in this study is the Myoshirt, a cable-driven device that provides gravity assistance to the shoulders, further detailed in Section 2.2. This device is a research prototype being developed by the Sensory-Motor Systems Lab at ETH Zürich. This study aimed first to assess the influence of the Myoshirt on the ROM of the shoulder and elbow joints, which is vital for performing daily tasks (Magermans et al., 2005). Second, the study examined the impact of the Myoshirt on endurance during a shoulder flexion task. Fatigue is a common challenge for individuals with UL impairments, limiting their ability to perform sustained activities (Severijns et al., 2018). Third, the study explored the effect of the exosuit on ADL task execution. This involves evaluating changes in perceived task difficulty as reported by the participants and the quality of task execution as rated by a therapist. Understanding these aspects provides insights into the device’s functionality in future potential real-world contexts.

To address these study goals, the following three hypotheses were investigated:

Hypothesis 1: Participants will demonstrate improved reachable workspace of the hand and joint ROM in the shoulder and elbow when partially supported by the Myoshirt compared to without the device.

Hypothesis 2: Participants will exhibit increased endurance during a shoulder flexion task when partially supported by the Myoshirt compared to without the device.

Hypothesis 3: Participants will perform ADL tasks with reduced perceived difficulty and increased observed quality when partially supported by the Myoshirt compared to without the device.

Qualitative feedback will be collected through questionnaires and verbal comments during the study. This will provide insights into the acceptance and usability of the Myoshirt.

## 2. Materials and methods

### 2.1. Participants

A total of nine participants were recruited through clinical collaborators in Switzerland, ensuring a heterogeneous representation of UL impairments. Specifically, six participants were recruited from the cereneo Center for Neurology and Rehabilitation (cereneo), two from Balgrist University Hospital, and one from Rehaklinik Bellikon. Recruitment was done by a contact person from each clinic, and all patients were tested on-site at their respective clinics. An in-depth description of the particular pathologies observed in this study, and the specific challenges they create, is provided in Section 1 of the Supplementary Materials.

In general, the inclusion criteria required impairments of the shoulder, particularly affecting shoulder flexion or abduction, from a neurological pathology. Individuals needed to have a passive ROM of at least 90° in shoulder flexion and abduction, along with neutral internal and external rotation, and full passive ROM at the elbow without pain. They also had to report the ability to sit for extended durations (1.5–2 hr) without discomfort and be free from severe pain or inflammation in the upper body and spine. Cognitive deficits were considered, and only those with mild or no impairment were considered, ensuring their ability to consent and understand the task instructions. Additionally, participants could not have severe shoulder subluxation or motor coordination impairments, such as those associated with brainstem or cerebellar lesions.

Specifically for stroke survivors, eligibility was restricted to adults who had experienced a first-time chronic stroke. Participants were required to have a Fugl-Meyer Upper Extremity motor score between 10 and 50. They were required to have no more than minimal sensory loss in the paretic arm and paresis confined to one side. Finally, spasticity had to be minimal, with a Modified Ashworth Scale score of three or less (Dunning, 2018).

All participants were assessed by therapists from their respective clinics to confirm their eligibility based on these criteria. During recruitment, potential participants were informed, along with information about the study, that it would not provide direct therapeutic benefits but could contribute to the development of future assistive technologies.

### 2.2. The Myoshirt

**Figure 1.**
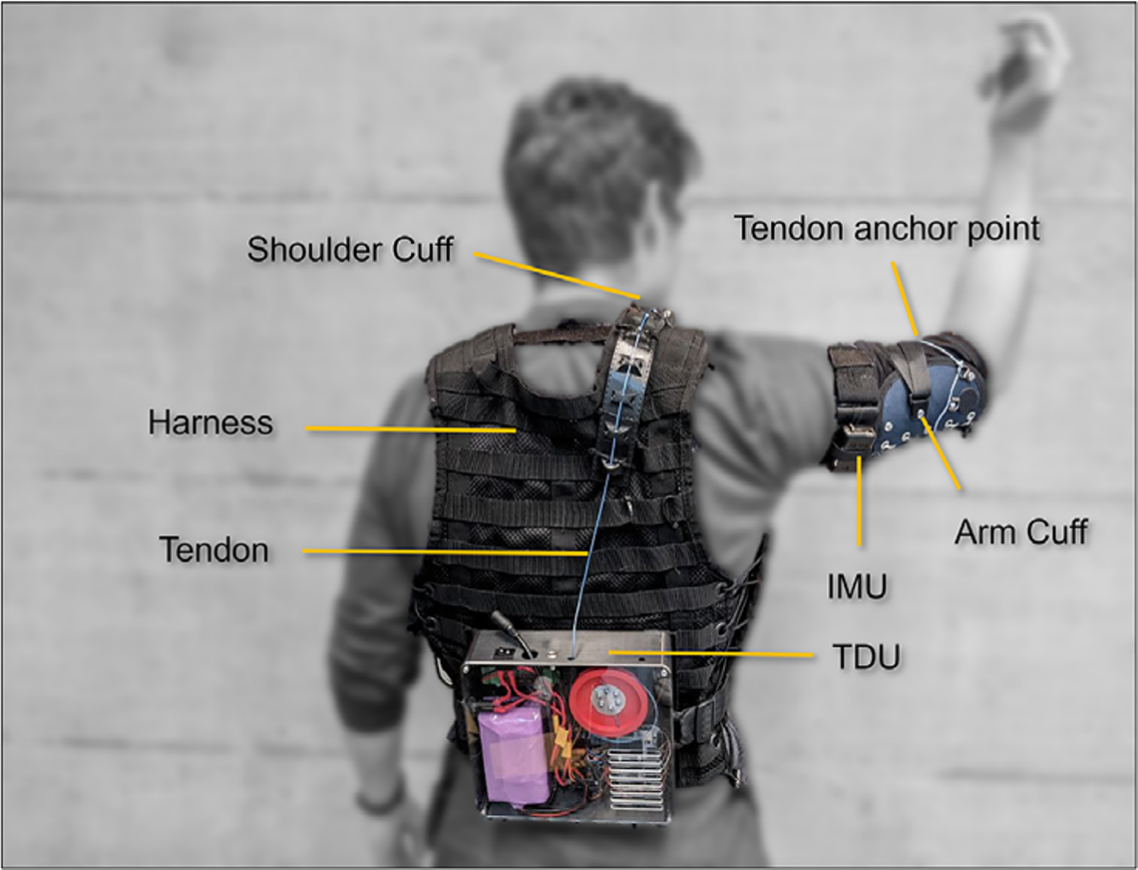
The Myoshirt as seen from the back of a user. On the back is the tendon driver unit (TDU). The cable runs up from the back of the user, through a shoulder cuff with redirect pulleys, and attaches to an arm cuff attached around the upper arm. The IMU on the upper arm relays poses information of the upper arm to the TDU to compute the appropriate cable tension. The amount of gravity supporting the system is specified in software as a percentage ranging from 0 to 100%, with 100% meaning that the system attempts to compensate for the entire gravity torque of the arm. The gravity torque of the arm is estimated through anthropometrics using the height and weight of the user.

The Myoshirt is a wearable exosuit, designed to provide active support for shoulder and upper-arm movements through gravity compensation. A first stationary version demonstrated the efficacy of providing gravity support to the shoulder joint in order to support diverse activities of the UL (Georgarakis et al., 2019; Georgarakis et al., 2020; Georgarakis et al., 2022). Later, a simplified portable version of the exosuit building off of this concept was developed and validated with healthy participants (Bardi et al., 2024; Esser et al., 2025). An overview of the functional components and operating principle of the Myoshirt is provided in Figure 1, and a more detailed description of the system is provided in Section 2 of the Supplementary Materials. The control scheme of the Myoshirt is detailed in Bardi et al. (2024). This is the first study using the Myoshirt on a broad patient group.

The donning of the Myoshirt is performed in a few steps. First, the vest is worn. This process is similar to putting on a knapsack (i.e., inserting both arms through the arm holes), then it is zipped up from the front. Second, the upper-arm cuff is slid over the hand, up the forearm, then secured by two velcro straps around the bicep. Then, the inertial magnetometer unit (IMU) is strapped over the arm cuff. Finally, the tendon exiting from the shoulder cuff is clipped into the arm cuff. Doffing of the Myoshirt involves the same steps performed in reverse order. All participants received support with the donning and doffing process from authors A.E. and F.M.

### 2.3. Optical motion capture system and markers

**Figure 2.**
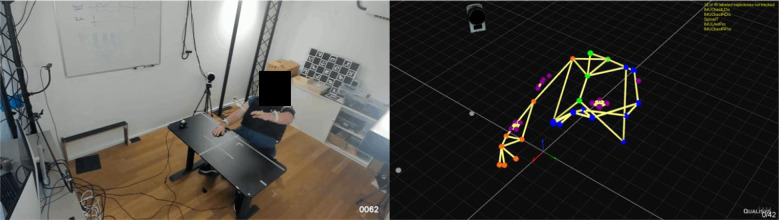
Side-by-side comparison of a measurement and the corresponding visualization of the marker set in Qualisys Track Manager.

At cereneo, data were collected using a Qualisys system (sampling frequency: 100 Hz, eight cameras), while at Balgrist University Hospital, a Vicon system (sampling frequency: 200 Hz, 26 cameras) was used. Reflective markers were placed on anatomical landmarks whenever feasible, provided these landmarks were not obstructed by the participant’s shirt or the Myoshirt, to ensure accurate tracking of trunk, arm, and hand movement 2. Hand markers were positioned proximally to the middle knuckle on both hands (metacarpophalangeal joint and dorsum of the hand). For the wrist, markers were attached to the radial and ulnar styloid processes. Elbow markers were positioned on the lateral and medial epicondyles. Shoulder markers were placed on the acromion process, while chest markers were attached to the sternum and in the fossa jugularis (jugular notch of the sternum). A single marker on the forehead was also included. The Qualisys system at cereneo included RGB video, while for the Rehaklinik Bellikon and Balgrist, a separate camera (Panasonic HDC-SD5, 1920 × 1080 p, 30 fps) was mounted on a tripod to record the measurements (Figure 2).

### 2.4. Study protocol

**Figure 3.**
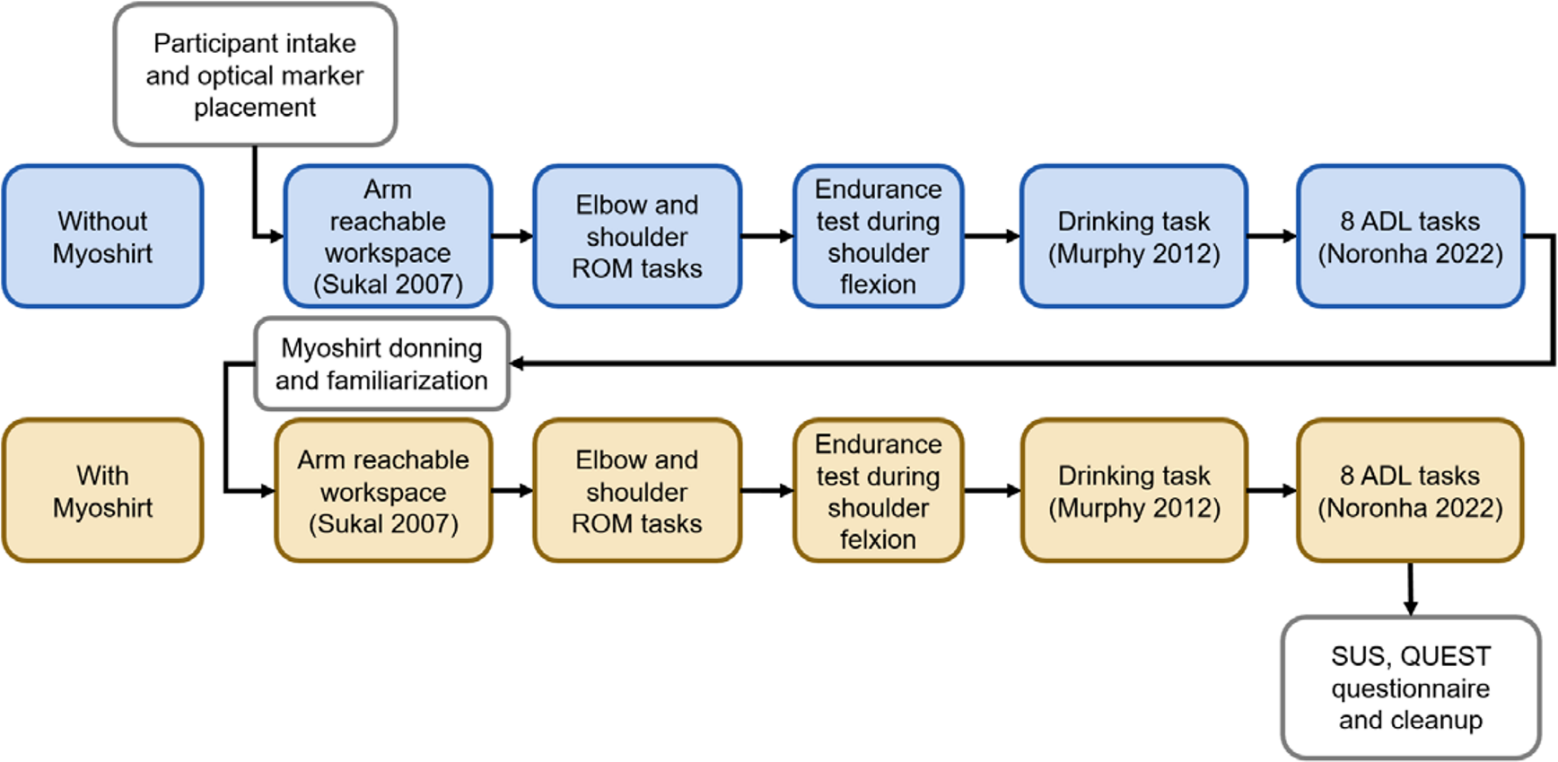
First, participants were welcomed, signed the informed consent, and underwent the process of placing the optical markers for motion capture. Then, participants underwent a series of tasks in a seated position, performed under two conditions: first, all the tasks were completed without the Myoshirt (**Without Myoshirt**), and then with the device (**With Myoshirt**). After the first condition, participants were assisted in donning the Myoshirt and underwent a familiarization phase to adjust to the device and select their preferred support level. During the familiarization phase, the participants started with 0% support (the minimum tension to keep the cable from going slack), and then the support was increased in 10% increments. At each point of increase, the participant was allowed to move their arm a bit and feel the support. At 40%, they could decide to keep it there, or drop it down to 30% if comfort was an issue. The support was capped at 40%, as it was found in previous work that short periods at 50% support already caused moderate shoulder discomfort in healthy participants (Bardi et al., 2024). The tasks were executed with the affected arm or, in cases of bilateral impairment, with the arm that the participant reported as more severely affected. Due to the small sample, the order of conditions was fixed. The With Myoshirt condition was performed last, so that any accumulated fatigue effects impact the exosuit condition and not the reference condition. Finally, the usability questionnaires were administered, and the participant was thanked for their time and given the Biberli cookie used in the study.

The data collection took place in a single session lasting 1.5–2 hr at one of the collaborating clinical sites: cereneo Hertenstein, Balgrist University Hospital, or Rehaklinik Bellikon. The study was structured as a cross-sectional experiment with two conditions performed in fixed order by each participant (3). The nonrandomized order was selected to prioritize participant comfort and safety throughout the protocol (Figure 3).

#### 2.4.1. Participant intake and preparation

Before the arrival of the participant, the study materials for the ADL tasks were prepared, and the optical motion capture (OMC) system was started and calibrated. Personal information, such as height, weight, and impairment specifics like the subjective level of impairment, was recorded for data analysis (Table 1). This was then followed by the placement of the reflective OMC markers.

**Table 1.** Participant characteristics

Participant ID	Age (years)	Height (m.)	Weight (kg)	Sex	Pathology	Subjective impairment level	Support level (%)
001	67	1.64	90	F	MS	Moderate	–
002	66	1.96	104	M	Stroke	Mild	30%
003	91	1.87	60	M	Stroke	Severe	40%
004	–	1.78	65	M	BPI	-	40%
005	61	1.75	119	M	Stroke	Severe	40%
006	69	1.63	84	F	-	Mild	30%
007	48	1.84	82	M	LDM	Severe	40%
008	50	1.81	85	M	BPI	Severe	40%
009	62	1.35	35	F	Polio	Severe	30%

Participant ID	Dominant side	Affected side	Tested side	Time since impairment	Location
001	R	L	L	7 years	cereneo
002	R	L	L	3 months	cereneo
003	R	L	L	4 months	cereneo
004	–	L	L	-	Bellikon
005	R	L	L	16 months	cereneo
006	R	Both	L	20 months	cereneo
007	R	Both	L	20 years	cereneo
008	R	R	R	10 years	Balgrist
009	R	Both	R	60 years	Balgrist

*Note.* Dashes indicate information that was not obtained from the participant or clinic.

Abbreviations: BPI, brachio-plexus injury; LDM, Laing distal myopathy; MS, multiple sclerosis.

#### 2.4.2. Hand reachable area

The hand reachable area was assessed using OMC. Participants were instructed to draw the largest possible circles with the affected arm from a seated hand placed above just off the table (Sukal et al., 2007; Ellis et al., 2016). Three circles were made in the clockwise and then another three in the counterclockwise directions.

#### 2.4.3. ROM tasks

ROM was assessed using OMC for the elbow and shoulder joint. For the shoulder, maximal active abduction and flexion were performed three times each. For the elbow, maximal ROM was assessed at three shoulder abduction configurations: 45°, 90°, and 135°. At each configuration, three repetitions of full elbow ROM were performed.

#### 2.4.4. Endurance test

An endurance test followed, in which participants elevated their affected arm to a shoulder flexion of 90°, or as high as possible if they could not, and held the position until volitional fatigue. The task was considered over once the arm dropped 10° below the initial elevation height. The duration was recorded using a stopwatch (and validated with OMC). As this test went until volitional fatigue, it was only performed once for each condition.

#### 2.4.5. Functional drinking task

Participants then performed a standardized drinking task, which involved reaching for a cup, grasping it with the affected hand, bringing it to the mouth, and returning it to the table (Alt Murphy et al., 2012). Kinematic movement quality metrics established in previous studies were used as outcome measures (Alt Murphy et al., 2018; Unger et al., 2024; Unger et al., 2024).

#### 2.4.6. ADL tasks

**Figure 4.**
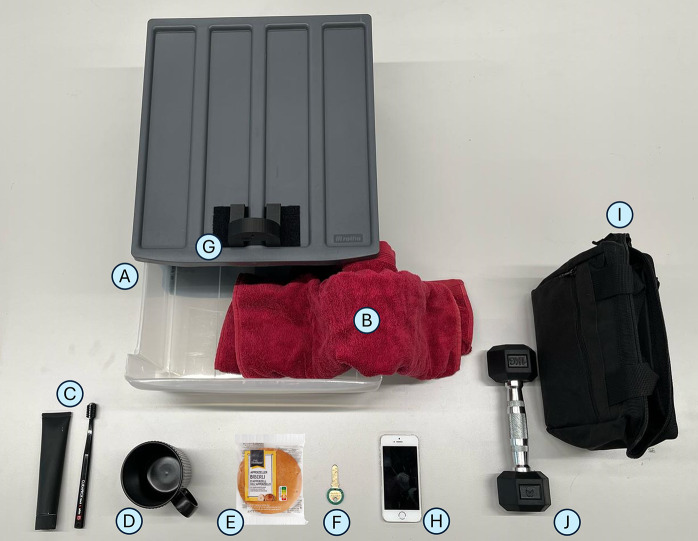
Materials for the ADL tasks. On top: a drawer (A) with a red towel (B). On the bottom from left to right: a toothbrush and toothpaste (C), a black cup (D), a packaged Biberli (E), a key (F) (3D-printed keyhole on top of the drawer, G), a smartphone (H), and a black bag (I) with 1 kg weight (J). The weight inside the bag during testing.

To evaluate ADL performance, participants attempted a series of eight tasks based on Noronha et al. (2022) (Figure 4). Each task was repeated three times by the participant when possible. All tasks from Noronha et al. were adapted as needed to be performed in a seated position, and two tasks (“chair” and “cupboard”) were omitted to ensure accessibility for wheelchair users among the participants. The tasks were initiated by the participant with both hands on the table. The focus of these tasks lies in their practical relevance and the variety of movements they require to simulate real-world functional activities without being overly specific, and they include the following:

1. Opening an empty drawer with the affected hand. The drawer was reset after each attempt.
2. Opening a drawer with the hand of the unaffected side, retrieving a towel, and placing it on the table on the same side as the affected arm.
3. Grasping a toothbrush with the hand of the affected side, mimicking the application of toothpaste using the hand of the unaffected side.
4. Picking up a cup (360 g) by its handle with the hand of the affected side, bringing it to the mouth, and holding it for 5 s.
5. Bringing a packaged Biberli (cookie) to mouth level and holding it for 5 s with the hand of the affected side.
6. Grasping a key with the hand of the affected side, bringing it up (30 cm above the table), and inserting it into a keyhole.
7. Grasping a phone (iPhone SE 2016, no case, 144 g) with the hand of the affected side, bringing it to the ear on the same side, and holding it for 5 s.
8. Picking up a bag by the handle (300 g) with 1 kg weight from the side of the chair on the affected side and lifting it onto the top of the drawer on that side (30 cm above table height). If not possible, place it only on the able (74 cm from the ground).

#### 2.4.7. Participant feedback on perceived difficulty

At the conclusion of each set of task, participants were asked to provide a perceived difficulty score on a 5-point Likert scale. A single rating was given for all repetitions of each task. This scale ranged from 1, meaning the task felt very easy or natural, to 5, indicating it was very difficult. This scale allowed the participants to share their impression of the subjective difficulty of a task under the respective condition. The difficulty rating without the Myoshirt was subtracted from the difficulty rating with the Myoshirt, thus a positive difference indicates that the Myoshirt made the task feel easier for the participant.

#### 2.4.8. Therapist feedback on perceived movement quality

In addition to participant self-reported measures, a physical therapist (author J.M.) assessed the quality of movement for all ADL tasks. The reviews were done with video recordings of the assessments after the experiment (Sauerzopf et al., 2024). A single rating was given for all repetitions of each individual task. Assessment was done via analysis of video recording, and the therapist did not know the diagnoses and only watched the ADL trials to avoid bias from observing other motions. This perspective complements the subjective measures for a comprehensive understanding of the impact of the Myoshirt on functional performance. The physical therapist used a 5-point Likert rating scale to evaluate the extent of compensatory movements used by participants. The ratings range from 1 to 5, where 1 indicated task completion with minimal or no compensatory movements and a healthy appearance, while 5 signified that the task could not be performed at all. The movement quality rating without the Myoshirt was subtracted from the rating with the Myoshirt; thus, a positive difference indicates that the Myoshirt improved the movement quality of the participant as perceived by the therapist.

#### 2.4.9. Postassessment feedback

At the end of the session, participants completed two standard questionnaires: the system usability scale (SUS; Brooke, 1996) and the Quebec User Evaluation of Satisfaction with Assistive Technology 2.0 (QUEST 2.0; Demers and Weiss-LambrouRand Ska, 2002). Participants were also encouraged to provide qualitative feedback regarding their experience with the Myoshirt.

### 2.5. Data collection and analysis

For the participant at Rehaklinik Bellikon (participant 004), an OMC system was unavailable. Thus, assessments were conducted visually, and ROM was recorded using a goniometer. For this reason, the data for this participant is reported separately.

#### 2.5.1. OMC data cleaning and labeling

All collected motion capture data, including that from the Vicon system used at Balgrist, were processed using Qualisys Track Manager (QTM). For gap filling, the methods used in order of usage preference were relational, polynomial, and finally linear filling.

#### 2.5.2. Processing methodologies

For the ROM tasks, shoulder flexion and abduction and elbow flexion/extension angles were calculated frame by frame, and peaks and troughs (representing maximum and minimum angles) were detected and averaged afterward from the three repetitions the participants completed during each task.

##### 2.5.2.1. Hand reachable area task: Enclosed area calculation

For the reachable work area task, the enclosed area of each circle was calculated via the shoelace formula:(2.1)

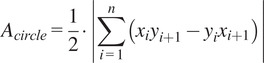



where 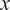 and 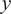 represent the spatial coordinates of the position of the hand after projection onto the surface of the table. The origin of this coordinate system for the motion capture data is the midpoint of the front of the table. This formula enabled the computation of the total work area for each participant, with results averaged across all circles to account for variations. Changes in work area with and without the Myoshirt were evaluated as a percentage difference, with positive percentage changes indicating an increased area with the Myoshirt compared to without.

##### 2.5.2.2. Joint angle rstimation

The marker coordinates allowed for the estimation of joint angles. For example, elbow angles were calculated using vectors formed by the shoulder, elbow, and the midpoint of the wrist markers. The angle between these vectors was determined simply using the dot product:(2.2)

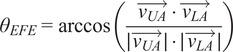



where 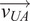 represents the vector from the shoulder to the elbow, while 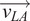 is the vector from the elbow to the midpoint of the wrist markers. The shoulder angles were computed in a similar fashion.

##### 2.5.2.3. Shoulder angles

A gravity-aligned vector was used as a fixed vertical reference for calculating the shoulder angle, as obstruction of the Myoshirt did not permit a standard biomechanical UL model to be used.

### 2.6. Statistical evaluation

The statistical evaluation focused on analyzing the effects of the Myoshirt on ROM, endurance, difficulty ratings, and execution quality.

All data were tested for normality using the Shapiro–Wilk test to determine the appropriate statistical tests. For paired comparisons between conditions (**With Myoshirt** versus **Without Myoshirt**), the data were analyzed using one-tailed paired t-tests if the data were normally distributed, as the hypotheses were directional. This approach is mathematically equivalent to performing a one-tailed one-sample t-test on the differences. When normality was violated, the Wilcoxon signed-rank (WSR) test was applied as a nonparametric alternative.

For single-condition analyses, such as testing percentage changes against a baseline, the same participant-specific differences or percentage changes were tested using one-tailed one-sample t-tests for normally distributed data, with the WSR test serving as the nonparametric alternative. For ordinal data, such as perceived difficulty and execution quality, WSR was used to evaluate if median changes between conditions were greater than zero.

All statistical evaluations were performed in Python, utilizing the scipy.stats library for implementing the Shapiro–Wilk test, paired t-tests, one-sample t-tests, and WSR tests.

## 3. Results

### 3.1. Participant characteristics

A total of nine participants were included in this study (Table 1). Participant 001 did not complete the study and was thus excluded from the analysis. The ROM results for Participant 004 are excluded from the statistical analysis and reported separately in Section 3 of the Supplementary Materials, as a goniometer was used for evaluation.

### 3.2. Range of motion

#### 3.2.1. Hand reachable area

**Figure 5.**
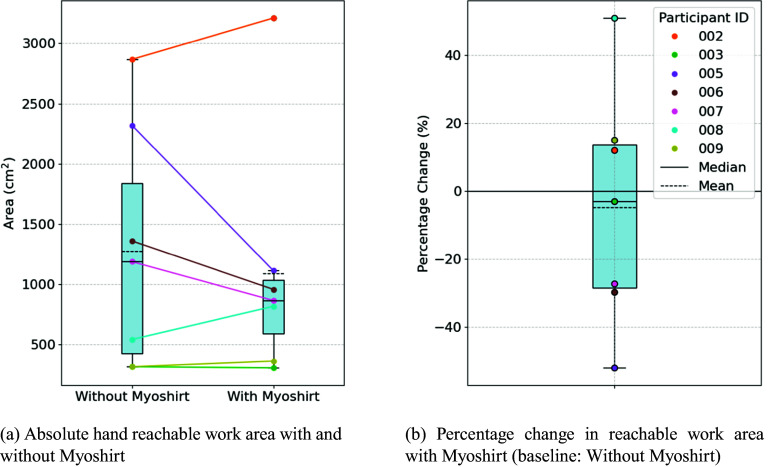
(a) The average maximum voluntary circle areas of the participants. (b) The percentage change between the two conditions. A larger positive percentage change means that the participant made larger circles with the Myoshirt, compared to without the Myoshirt.

The reachable work area, measured during circular arm movements, was analyzed under the **Without Myoshirt** and **With Myoshirt** conditions. The average reachable area was 1,271 cm^2^ without the Myoshirt and 1,088 cm^2^ with the Myoshirt (Figure 5a). No analysis was performed on the absolute areas because they are not normalized; thus, participants with longer or shorter arms will also make larger or smaller circles accordingly. For the percentage changes, on average, the reachable area decreased by 4.97% when the Myoshirt was used (Figure 5b). The percentage differences were normally distributed (W = .889, p = .267), and no significant increase was observed (t(6) = −.381, p = 1.000).

#### 3.2.2. Shoulder abduction

**Figure 6.**
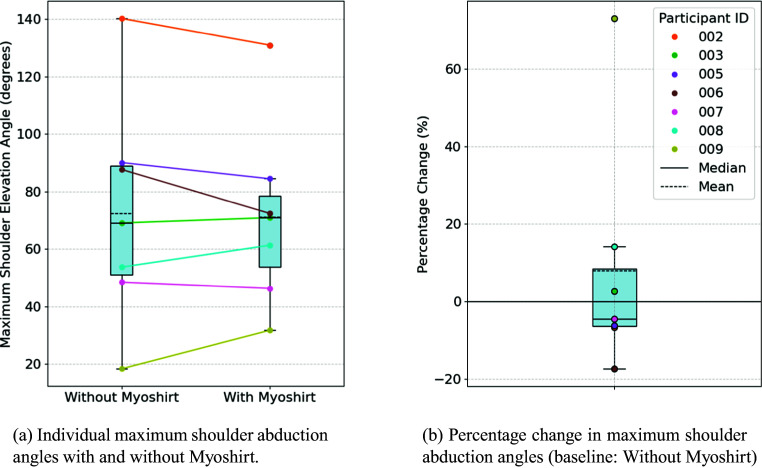
(a) The maximum shoulder abduction angle with and without the Myoshirt.(b) the percentage change between the two conditions. A larger percentage change indicates that the participant could abduct the shoulder higher with the support of the Myoshirt.

The average maximum angle achieved without the Myoshirt was 72.5°, compared to 71.1° with the Myoshirt (Figure 6a). Statistical analysis indicated no significant difference between the two conditions. Normality of the absolute differences was confirmed (W = .990, p = .993), and no significant increase in maximum shoulder abduction angles with the Myoshirt was observed (t = −.366, p = 1.000).

The average percentage change across participants was 7.87% (Figure 6b). The percentage differences were not normally distributed (W = .741, p = .010), and no significant differences were found (W = 3.0, p = .594).

#### 3.2.3. Shoulder flexion

**Figure 7.**
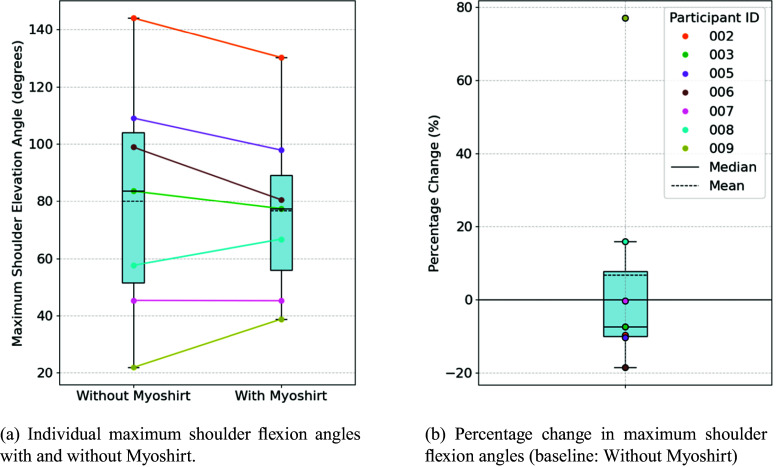
(a) The maximum shoulder flexion angle with and without the Myoshirt. (b) The percentage change between the two conditions. A larger percentage change indicates that the participant could flex the shoulder higher with the support of the Myoshirt.

The average maximum angle achieved without the Myoshirt was 80.0°, while the average with the Myoshirt was slightly lower at 76.6°, resulting in an average absolute difference of −3.38° (Figure 7a). The absolute differences were normally distributed (W = .948, p = .708), and no significant increase in shoulder flexion angles with the Myoshirt was observed (t = −.700, p = 1.000).

The average percentage change across participants was 6.70% (Figure 7b). The percentage differences were not normally distributed (W = .732, p = .008), and the change was not significant (W = 12.0, p = .656).

#### 3.2.4. Elbow flexion and extension

**Figure 8.**
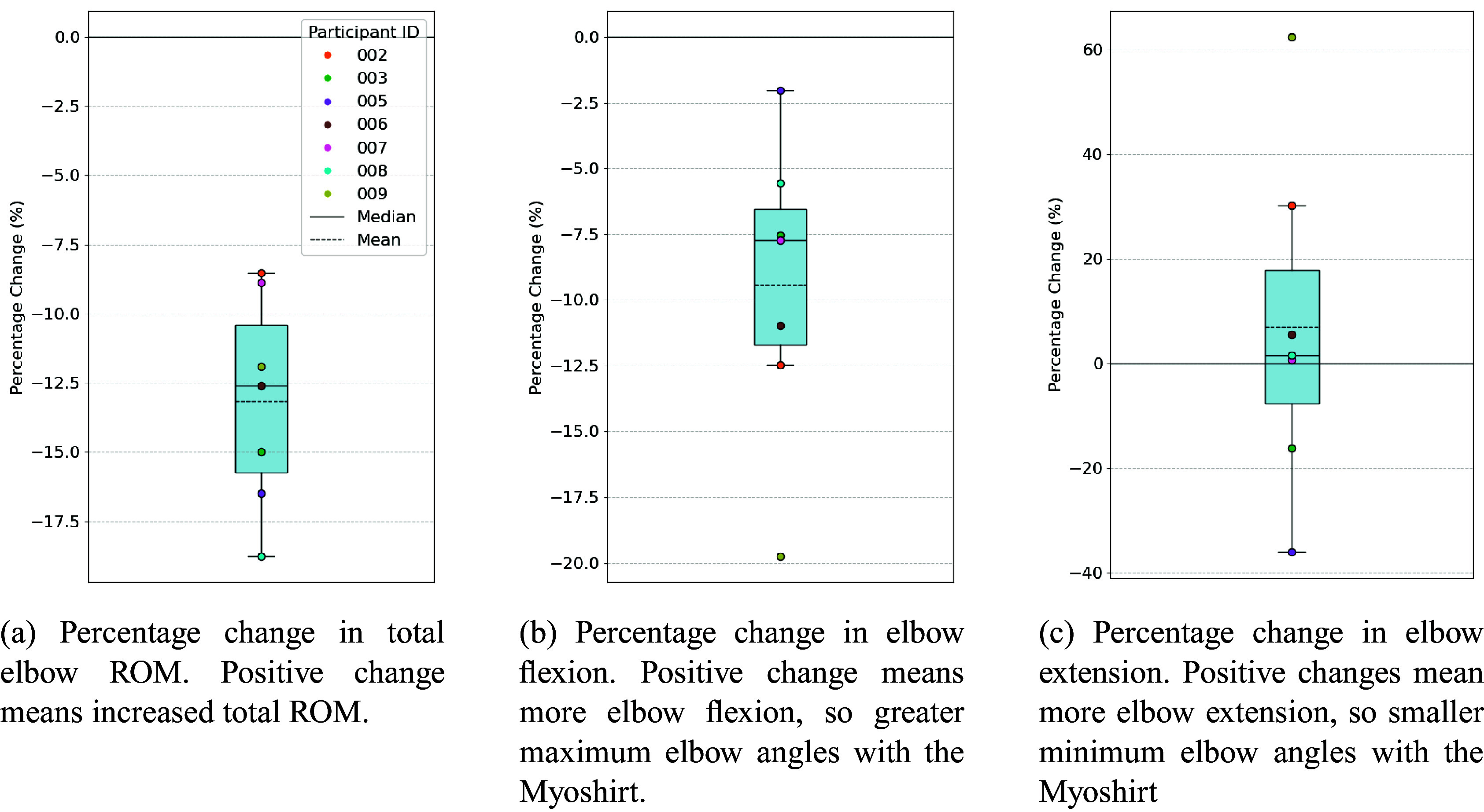
The percentage change in total elbow ROM (a), elbow flexion (b), and elbow extension (c). The graphs are all arranged so that positive percentage changes indicate an increasing ROM.

The average total ROM without the Myoshirt was 97.9°, while the average with the Myoshirt was 86.0°, resulting in an average absolute difference of −11.8° and an average percentage decrease of −13.2% (Figure 8a). The differences in total elbow ROM were normally distributed (W = .844, p = .108). No significant increase in elbow ROM with the Myoshirt was observed (t = −6.078, p = 1.000). For the percentage differences, normality was also confirmed (W = .951, p = .737), and no significant increase was observed (t = −6.078, p = 1.000).

The average maximum angle (maximum flexion) without the Myoshirt was 126°, while the average with the Myoshirt was 113°, resulting in an average absolute difference of −13.0° and an average percentage decrease of −9.43% (Figure 8b). These differences were normally distributed (W = .910, p = .396). No evidence supporting an increase in maximum elbow angles with the Myoshirt was observed (t = −3.361, p = 1.000). Similarly, for the percentage differences, normality was confirmed (W = .955, p = .772), and no significant increase was observed (t = −3.361, p = 1.000).

The average minimum angle without the Myoshirt was 28.2°, while the average with the Myoshirt was 27.1°, resulting in an average absolute difference of −1.13° and an average percentage decrease of −6.83%, (Figure 8c). The differences were normally distributed (W = .976, p = .936). No significant reduction in minimum angles was observed (t = −.334, p = .375). For the percentage differences, normality was confirmed (W = .953, p = .761), and no significant reduction was observed (t = −.334, p = .375).

Data collected during additional tasks at 90° and 135° shoulder abductions were not included in the analysis, as only three participants completed the 90° task and one completed the 135° task, rendering the sample size insufficient for meaningful evaluation.

### 3.3. Endurance

**Figure 9.**
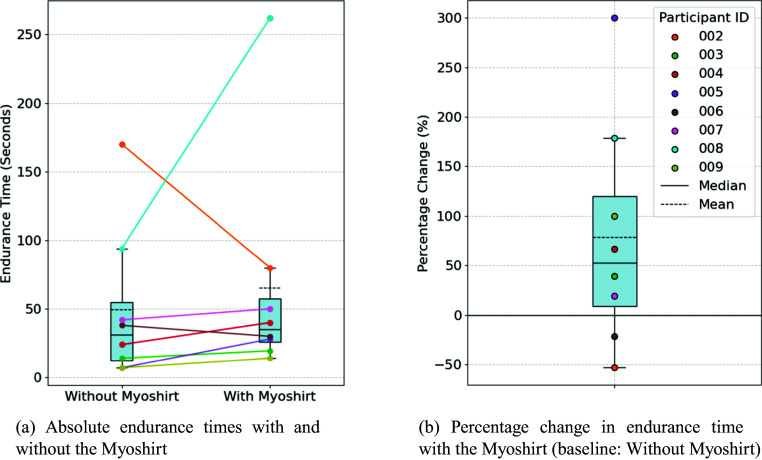
(a) The absolute times during the forwards flexion endurance task, without and with the Myoshirt. (b) The percentage change between the two conditions, where a positive change indicates that the arm could be help up longer with the Myoshirt.

On average, participants maintained the arm elevation for 49.5 s without the Myoshirt and 65.4 s with the Myoshirt, resulting in an absolute increase of 15.9 s (Figure 9a). The absolute time differences were not normally distributed (W = .900, p = .025). The statistical test showed no significant difference in absolute endurance (W = 25.5, p = .191). Endurance time increased by 78.7% (Figure 9b). The normalized time differences were normally distributed (W = .971, p = .516), and a marginally significant increase was observed (t = 1.942, p = .047). With the positive outlier (P005) removed, the test becomes nonsignificant (t = 1.63, p = .077).

### 3.4. Drinking task kinematics

The metrics proposed by Alt Murphy et al. (2018) were analyzed, and the elbow extension angle during the reaching phase of the drinking task showed a significant positive change from baseline (n = 5, only cereneo participants). On average, elbow extension increased by 13.5% (t = 7.52, p = .002) during the reaching phase of the drinking task.

### 3.5. Activities of daily living

#### 3.5.1. Perceived difficulty

**Figure 10.**
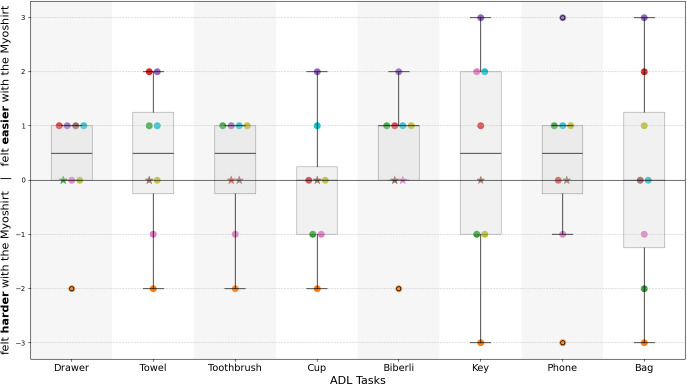
Subjective difficulty differences for ADL tasks with and without the Myoshirt. The y-axis represents the difference in difficulty ratings computed by subtracting the score for the **Without Myoshirt** condition from the score for the **With Myoshirt** condition; thus, positive values indicate tasks felt easier with the Myoshirt. The participants are indicated by colored circles, and the boxplot whiskers indicate the interquartile range, while the black line represents the median score. The star symbol in the graph represents tasks rated as very easy (score of 1) without and with the Myoshirt, indicating a floor-effect, as participants could not report perceived improvements with the Myoshirt for these tasks due to the scale’s constraints.

The perceived difficulty ratings of most tasks were slightly reduced when supported by the Myoshirt, with a median difference of .5 (Figure 10). The overall median differences across all tasks decreased (W = 21.000, p = .010), indicating a significant reduction in perceived difficulty with the Myoshirt. Task-specific analysis revealed no statistically significant reduction for perceived difficulty in individual tasks, with p values ranging from .140 to .609.

When analyzing participant-level median differences across all tasks, the data showed a median difference of .5. However, this analysis did not reveal a statistically significant reduction in difficulty (W = 17.500, p = .275). Finally, when all task ratings were analyzed collectively, the overall median difference was .0. The combined differences also did not indicate a significant reduction in perceived difficulty (W = 668.500, p = .127).

#### 3.5.2. Execution quality rating by therapist

**Figure 11.**
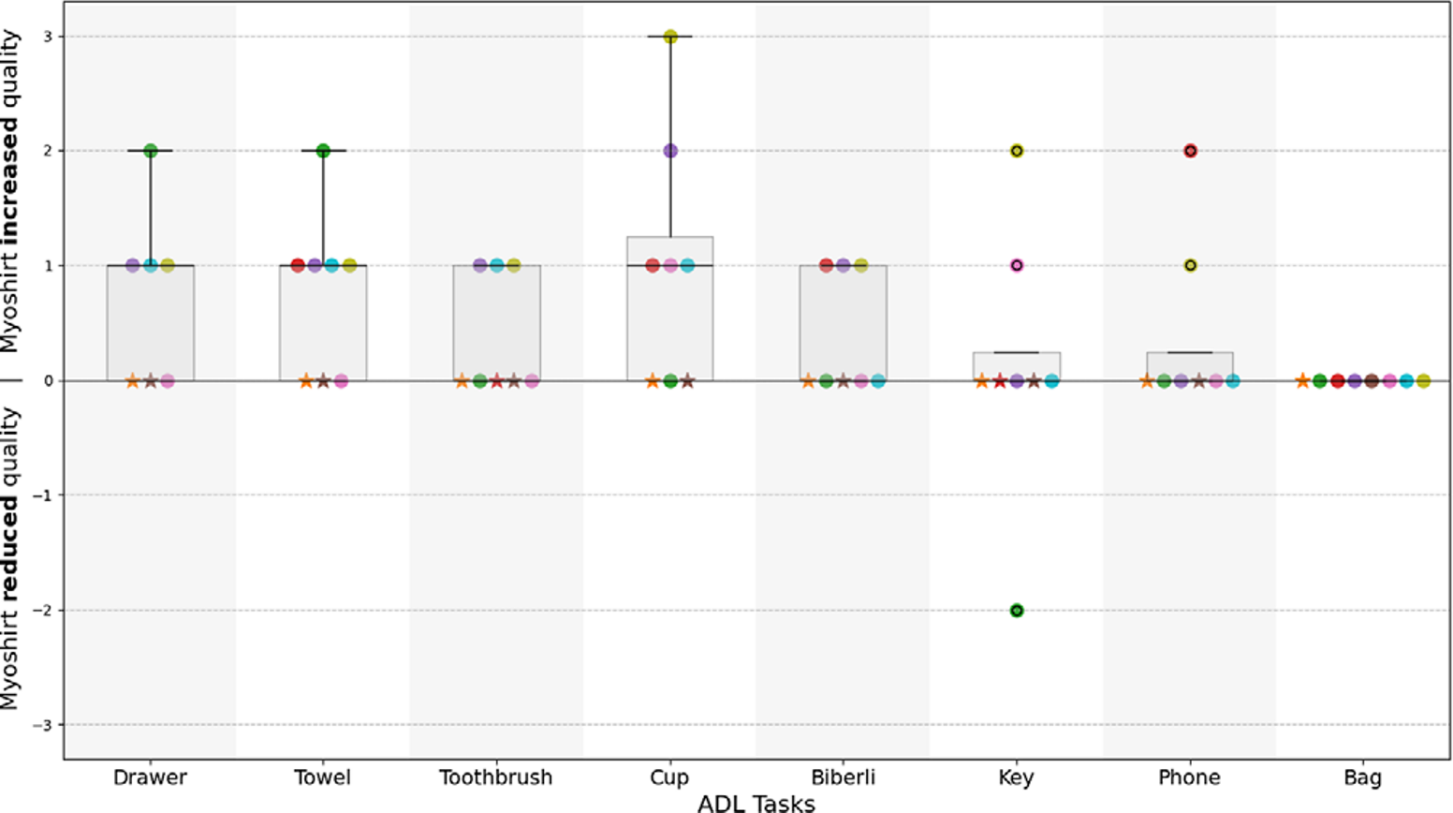
Therapist-rated quality of task execution with and without the Myoshirt, based on observed compensatory movements and execution quality. The y-axis represents the difference in movement quality ratings computed by subtracting the score for the **Without Myoshirt** condition from the score for the **With Myoshirt** condition; thus, positive values indicate that tasks were performed with better movement quality with the Myoshirt. The participants are indicated by colored circles, and the boxplot whiskers indicate the interquartile range, while the black line represents the median score.

The therapist’s quality ratings indicate an overall trend of improvement (reduction in compensatory movements and improvement in execution quality) in task execution when participants used the Myoshirt (Figure 11). Statistical analysis of execution quality ratings revealed a median difference of 1.0 for the drawer, towel, and cup task, and a median difference of .0 across all other tasks. A weak statistically significant improvement in execution quality across all tasks was observed (W = 6.000, p = .042), with a small sample size (*n* = 8) contributing to the lack of statistical power. When analyzing participant-level median differences, the data similarly showed a median difference of .25, with a slightly statistically significant improvement (W = 10.000, p = .029). Task-specific analysis revealed statistically significant increases for the drawer (median = 1, W = 10.000, p = .029), towel (median = 1, W = 15.000, p = .017), toothbrush (median = 0, W = 6.000, p = .042), cup (median = 1, W = 15.000, p = .020), and Biberli (median = 0, W = 6.000, p = .042) tasks. Other tasks showed p values ranging from .090 to .393, with no evidence of significant improvements. Extensive supplemental notes on the scoring is provided in Section 5 of the Supplemental Materials.

### 3.6. System usability scale

**Figure 12.**
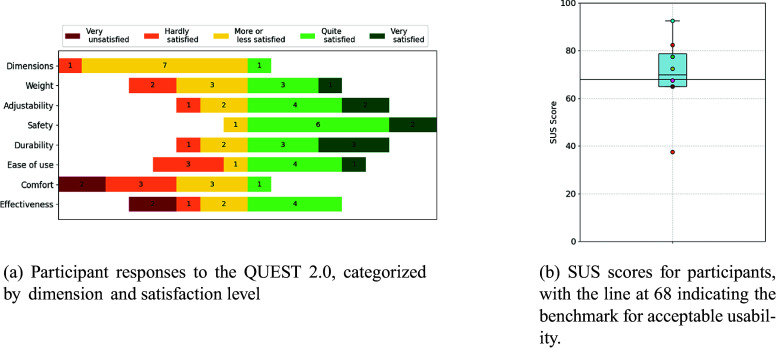
(a) The results from the QUEST2.0 survey.(b) The results of the SUS survey.

The mean SUS score across all participants was 68.6 out of 100, although individual scores as low as 38 and as high as 92 were observed (Figure 12b).

### 3.7. Quebec User Evaluation of Satisfaction with Assistive Technology 2.0

The results of the QUEST 2.0 show that participants were generally satisfied with the safety, durability, and adjustability of the Myoshirt, with most ratings in these categories falling in the “quite satisfied” or “very satisfied” range (Figure 12a). In contrast, comfort received lower ratings, with five participants indicating dissatisfaction (“very unsatisfied” or “hardly satisfied”). Ease of use showed mixed feedback, with four participants reporting they were “quite satisfied” or “very satisfied,” while three indicated lower satisfaction levels.

### 3.8. Qualitative Feedback

Qualitative comments obtained from the participants after the study are documented in Section 4 of the Supplementary Materials.

## 4. Discussion

**Figure 13.**
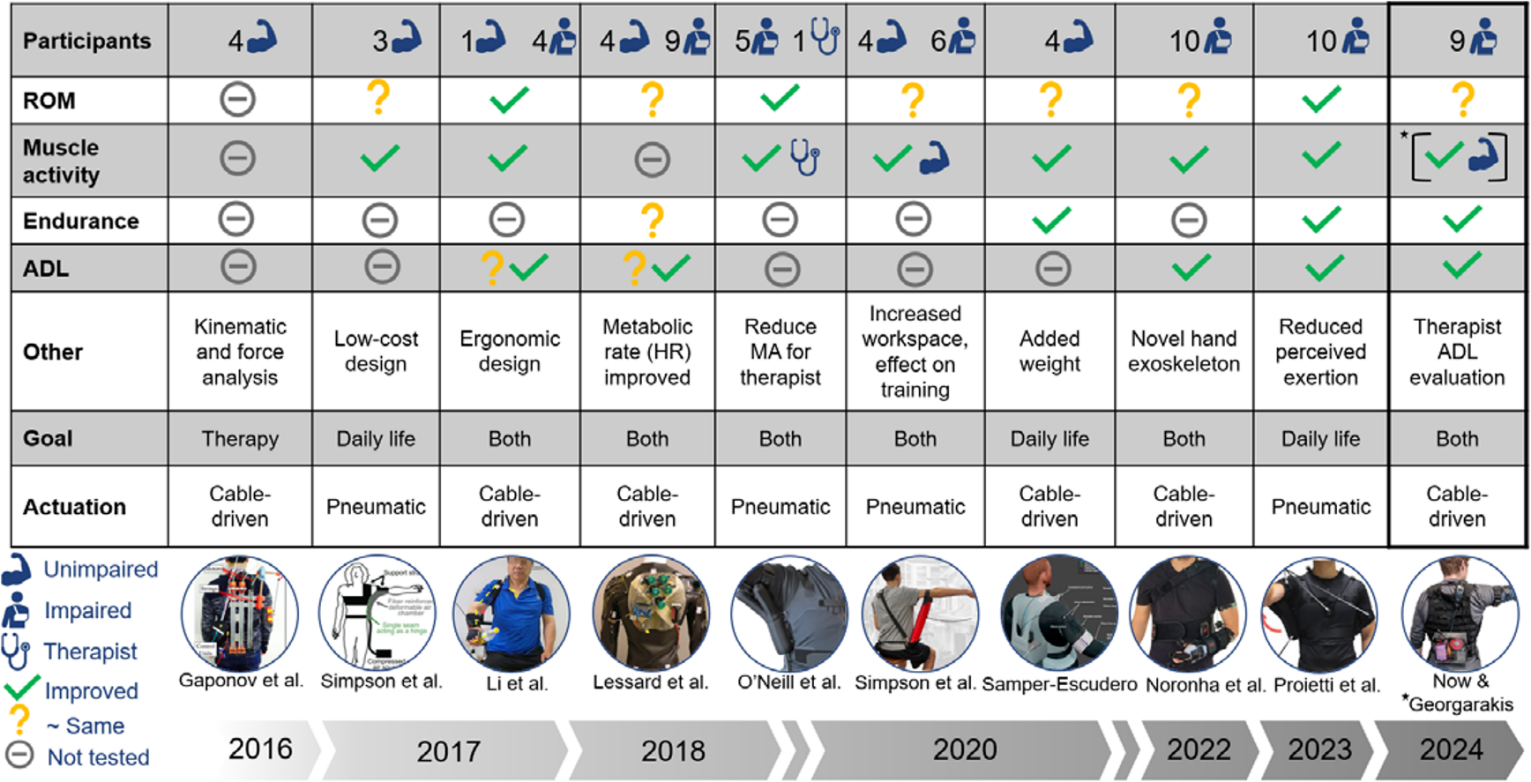
Summary of comparable devices and studies, including the Myoshirt. In the last column, the reduction in muscular activity was demonstrated in Georgarakis et al. (2022), hence the asterisk and brackets, while the other results in the column come from this work. Works include Georgarakis et al. (2022), Gaponov et al. (2017), Simpson et al. (2017), Li et al. (2018), Lessard et al. (2018), O’Neill et al. (2020), Simpson et al. (2020), Samper-Escudero et al. (2020), Noronha et al. (2022), and Proietti et al. (2023).

This study evaluated the Myoshirt, a cable-driven wearable assistive device designed to provide gravity compensation for the shoulders, aiming to enhance ROM and functionality in individuals with impairments. Many studies (see Figure 13 for a comparative overview) have been performed to understand the impact of various research exosuits on ROM, muscular activity, endurance, and performance of daily activities, which has been widely explored.

Hypothesis 1 could not be met, as the Myoshirt did not significantly increase ROM, a limitation also observed in some other devices. Hypotheses 2 and 3 were supported. The Myoshirt significantly increased endurance, consistent with findings from comparable systems. The device also reduced perceived task difficulty and enhanced execution quality in ADL tasks, aligning with improvements shown by other assistive technologies.

### 4.1. Range of motion

#### 4.1.1. Hand reachable area

The hypothesis that participants would demonstrate an improved reachable work area with the Myoshirt was not supported by the results. This contradicts the findings of other studies (Sukal et al., 2007; Simpson et al., 2020; Ellis et al., 2009). On average, the reachable work area decreased slightly when wearing the Myoshirt, and the statistical analysis did not reveal significant differences between the **Without Myoshirt** and **With Myoshirt** conditions. This outcome suggests potential limitations of the device or confounding factors that may have influenced the results. Sukal specifically studied chronic stroke patients with elevation-flexor synergy; thus, it could be that the alleviation of flexor synergy allowed increased reachable areas with the hand, something not observed with the diverse patient group in this study.

#### 4.1.2. Shoulder abduction and flexion

For shoulder abduction and flexion, the results did not support **Hypothesis 1**, namely that the Myoshirt would enhance ROM. The visual inspection of Figures 6a and 7a shows a similar shape, where participants with higher elevation angles (without the Myoshirt) tend to reduce with the Myoshirt (e.g., Participants 002 and 006), while participants with lower maximum elevation angles (without the Myoshirt) tend to increase their elevation with the Myoshirt (e.g., Participant 009). For Participants 002 and 006, the already high baseline elevation values may have left minimal room for improvement, and the support of the Myoshirt could not provide additional benefits. Additionally, at higher shoulder angles, the shoulder cuff and padding of the Myoshirt blocked or restricted movement, further contributing to the observed reductions in ROM. For the more impaired participant 009, they could benefit from the support of the Myoshirt before reaching this limit.

These findings align with an earlier study using a prior version of the Myoshirt by Georgarakis et al. (2022). They reported a statistically significant reduction in ROM among healthy participants while showing no statistically significant ROM changes in participants with impairments. Similarly, other studies on assistive devices have struggled to demonstrate significant improvements in ROM (Simpson et al., 2017; Lessard et al., 2018; Simpson et al., 2020; Samper-Escudero et al., 2020; Noronha et al., 2022).

#### 4.1.3. Elbow flexion and extension

**Hypothesis 1** that the Myoshirt would improve elbow ROM during the 45° shoulder abduction task was not supported by the data. On the contrary, total ROM decreased slightly when using the Myoshirt. Our initial hypothesis stemmed from earlier findings suggesting that shoulder support could improve elbow ROM in individuals with flexor synergies in stroke patients (Sukal et al., 2007; Ellis et al., 2009). However, none of the stroke participants in this study exhibited flexor synergies, which may explain the absence of increased elbow ROM, as the Myoshirt does not support the elbow itself. Additionally, the inclusion of participants with diverse pathologies further diminishes the relevance of this mechanism to the observed outcomes.

A key factor contributing to the reduced ROM appears to be the significant reduction in maximum elbow angles, indicating restricted elbow flexion. The average maximum elbow angle was significantly lower with the Myoshirt, suggesting that the device could have hindered participants’ ability to achieve full flexion. This limitation is likely tied to the design of the upper arm cuff, which may have physically restricted movement during flexion. The tight fit of the cuff could have limited the biceps’ ability to expand during flexion, potentially hindering maximum flexion angles. Additionally, the cuff’s proximity to the elbow joint on the upper arm may have further obstructed the movement.

It is also worth noting that participants performed the elbow flexion-extension task at 45° shoulder abduction. Under these conditions, gravity naturally assists with elbow extension, potentially diminishing the device’s impact on this movement.

### 4.2. Endurance

The endurance test results indicate that the Myoshirt generally improved participants’ ability to maintain arm elevation and, therefore, support **Hypothesis 2** of expecting increased endurance when supported by the device. Relative changes provided a more meaningful perspective due to the variability in participants’ muscle strength, impairments, and baseline endurance levels. This aligns with findings from other studies on assistive devices, as shown in Figure 13, where endurance increased when using an assistive device.

Participants 002 and 006, who reported mild impairments, were the only ones whose endurance times decreased with the Myoshirt. This suggests that participants with fewer functional limitations may benefit less from the device or encounter factors that offset its supportive effects. For example, Participant 006 noted that they could have continued longer with the Myoshirt due to its gravity compensation but chose to stop earlier, underscoring the influence of individual motivation and perception during the task.

For participants with greater impairments, the gravity compensation of the Myoshirt appeared to reduce the physical burden of the arm, as reflected in their increased endurance times. This effect could be attributed to reduced muscle activity observed during an arm-raising task when supported by the Myoshirt, compared to the task being performed without the device (Bardi et al., 2024).

### 4.3. Drinking task kinematics

It was observed that during the reaching phase of the drinking task, participants consistently achieved higher elbow extension when supported by the Myoshirt (Section 3.4), a result that differs from the ROM assessment, where flexion was reduced and extension remained similar (Section 3.2.4). This could indicate that the support from the exosuit reduced forward trunk compensation and allowed for a healthier movement quality. The Myoshirt was developed to primarily support forward shoulder flexion and reaching actions, and this could be why the benefits for the elbow are seen during the drinking task but not the pure elbow ROM task (Georgarakis et al., 2019).

### 4.4. ADL tasks

The results provide weak support for **Hypothesis 3**, regarding the influence of the Myoshirt on ADL task performance. A significant overall reduction in perceived difficulty was observed across all tasks, suggesting a potential measurable benefit in reducing task difficulty. However, execution quality ratings by the therapist showed a significant effect only when analyzing all individual ratings collectively. This indicates that while trends of improvement in execution quality were present across the aggregated ratings, they were not consistently reflected in the central tendency of task- or participant-level medians.

Individual variability and task-specific effects remain notable. Participants with severe impairments appeared to benefit more from the Myoshirt than those with mild impairments, reinforcing its potential to address more substantial functional limitations. For example, Participants 002 and 006, who reported mild impairments, did not benefit from the Myoshirt, as reflected by their low perceived difficulty scores matching the therapist’s rating scores for every task under both conditions being executed normally and appearing healthy. On the other hand, Participants 005, 008, and 009 (with self-reported severe impairment levels) demonstrated consistent reductions in ADL difficulty when using the Myoshirt. These findings suggest that the Myoshirt may be less suitable for individuals with mild impairments, where the additional forces generated by the device could outweigh its benefits.

Although compensatory movements were reduced in some cases, this effect was not universal. Participant 003 exemplified a unique scenario where they assisted their affected arm with the unaffected arm while using the Myoshirt during the key task. This behavior, not observed without the device, led to a lower execution quality rating due to the introduction of compensatory strategies. Such findings highlight the complexity of evaluating movement quality and the interplay of individual factors.

While the Myoshirt demonstrates potential to temporarily enhance task performance for participants with severe impairments, its utility for individuals with mild impairments remains limited. The observed trends suggest that the Myoshirt may provide functional support in simulated real-life tasks, but further optimization of its design is essential to maximize its effectiveness across diverse user groups and investigate if these benefits extend to real-life tasks in unsupervised settings. Despite the different evaluations of ADL tasks, other assistive devices support these findings, as they were also able to show trends toward improvement in task execution when supported (Li et al., 2018; Lessard et al., 2018; Noronha et al., 2022; Proietti et al., 2023).

### 4.5. Questionnaires

The results from the SUS and the QUEST questionnaire both highlight the potential usability of the Myoshirt and indicate that there is room for improvement. The mean SUS score falls in the high marginal range of acceptability (Bangor et al., 2009), indicating “ok” to “good” usability and meaning that the usability of the Myoshirt is comparable to average systems evaluated with this tool. However, the variability in individual responses reflects diverse user experiences. Given that the SUS is a general-purpose tool not specifically designed for assistive devices, some usability issues identified may be expected for a prototype like the Myoshirt. However, the assistive device with pneumatic actuators used in a study by Proietti et al. managed to achieve an SUS score of 79.2 (Proietti et al., 2023), indicating better overall usability than the Myoshirt. While this makes SUS a valuable benchmark, it does not pinpoint specific aspects to address, which is where the QUEST results provide more actionable insights.

The QUEST ratings reveal generally positive feedback regarding safety, durability, and adjustability, which were rated as “quite satisfied” or “very satisfied” by most participants. In contrast, comfort and effectiveness emerged as significant areas of concern. Comfort was rated “very unsatisfied” or “hardly satisfied” by multiple participants, with most participants verbally mentioning that the control box on the lower back was uncomfortable when sitting down, and others reporting discomfort due to the cable of the Myoshirt exerting heavy pressure on their shoulders.

These results underscore again the importance of addressing individual needs in the design of the Myoshirt. Enhancing comfort, particularly by reducing pressure from the TDU and cables, as well as improving support functionality, especially for users requiring lower arm assistance, could significantly improve user satisfaction. While the positive feedback on safety, durability, and adjustability reflects the device’s strengths, targeted improvements in comfort and effectiveness are essential for broader user acceptance and usability in daily life.

### 4.6. Study limitations

The study’s limitations primarily stem from the broad inclusion criteria and methodological challenges, which impacted both data interpretation and possible comparisons. By including participants with varied impairment levels and pathologies, the sample size for specific subgroups was too small to allow meaningful statistical analyses or subgroup comparisons. Future studies should target better stratified groups. A self-selected support level could also be treated as a covariate in the statistical analysis (Runnalls et al., 2019). Time limitations from clinics prevented as well a third tested condition (exosuit in transparency mode). This would have also allowed the rating therapist to be blinded to the exosuit condition, and a statistical analysis that could decouple the passive effect of wearing the Myoshirt from the effect of the active support of the Myoshirt. Therapist evaluations of movement quality, though insightful, were limited to a single rater. Including multiple raters (who could also be blinded with a sham condition) would have enhanced the reliability and robustness of these assessments.

The use of markers placed on participants’ shirts and the Myoshirt, as visualized in Figure 2, presented significant challenges in accurately capturing joint angles. Marker placement on garments led to increased motion artifacts. Additionally, as the Myoshirt blocked key anatomical landmarks, a decision not to use a biomechanical model in the analysis was made (e.g., Plug-in Gait, Vicon, 2024). For example, an initial approach to computing the shoulder angle attempted to account for trunk movement in the coronal plane. This approach involved using the normal vector of the sagittal plane from two reference vectors formed by specific anatomical landmarks: one extending from the sternum to the fossa jugularis and another from the sternum to the C7 marker. The cross product of these vectors produced the normal vector to the sagittal plane, which was then dynamically aligned with an initial fixed vertical reference vector using a rotation matrix. Extraneous marker shifts, unrelated to actual compensatory movements and visible in the video recordings, had disproportionate effects on the results. Ultimately, a gravity-aligned vector was used as a fixed vertical reference for calculating the shoulder angle. This approach provided better consistency and comparability among conditions and participants, at the cost of a less accurate measurement of the shoulder angle.

Finally, the nonrandomized order of the conditions represents a methodological limitation, as accumulated fatigue and task learning could impact the results for the exosuit condition. By introducing the Myoshirt after the participants are comfortable with the baseline experimental protocol, it can be observed how participants react to the tasks without the additional variable of an unfamiliar device. The effect of fatigue was mitigated by pacing the session appropriately, providing breaks, and frequently checking in with the participant. Nevertheless, a randomized or counterbalanced order would have allowed for a more rigorous analysis of order-dependent effects and strengthened the validity.

### 4.7. Outlook

The study was very useful in driving some design decisions for the next iteration of the Myoshirt (fit, comfort, cuff interfaces, shoulder padding, TDU placement). Future research should explore the long-term impact of the Myoshirt through extended use in real-world settings (standing, cooking, cleaning) to assess its potential for fostering motor function recovery and improving daily living outcomes. Practice over multiple sessions could make incremental progress that compounds, accompanied by increased familiarity and comfort using the device. As well, an intervention/training study with semisupervised use of the device in the clinics may be of interest for future work.

## 5. Conclusion

The Myoshirt represents an innovative step toward developing wearable assistive technology designed to improve arm function for individuals with UL impairments. Through a combination of motion analysis, user feedback, and therapist evaluations, the findings highlighted both the promise of this technology and the areas that require further refinement.

While the Myoshirt provided measurable benefits, such as reducing perceived difficulty for specific tasks and improving execution quality as rated by a therapist, these benefits were not consistent across all participants. The benefits of the Myoshirt were most pronounced for individuals with more severe impairments.

While this study underscores the potential of the Myoshirt, it also highlights the challenges inherent in developing assistive devices that meet the complex needs of diverse user populations. By continuing to refine and evaluate the Myoshirt in collaboration with users and clinicians, this technology can move closer to fulfilling its promise of enhancing mobility, independence, and quality of life for individuals with UL impairments. The journey toward achieving this goal represents not only a technological challenge but also an opportunity to rethink how assistive devices can truly empower their users.

## Supporting information

Esser et al. supplementary material Esser et al. supplementary material

## Data Availability

Data can be made available to interested researchers upon request by email to the corresponding author.

## Supplementary material

The supplementary material for this article can be found at http://doi.org/10.1017/wtc.2025.10024.
