# Supplementary material for: Impact of a shoulder exosuit on range of motion, endurance, and task execution in users with neurological impairments
Source: Wearable Technol. 2025 Aug 11;6:e42. doi: 10.1017/wtc.2025.10024 (PMC12441642; doi:10.1017/wtc.2025.10024)
Supplement: Esser et al. supplementary material [file S2631717625100248sup001.pdf]

Impact of a shoulder exosuit on range of motion, endurance, and task execution in users with neurological impairments
Supplementary materials

1 Pathologies Present in Study

This section provides some more depth to the diverse pathologies observed in this study.

1.1 Stroke

Stroke is a leading cause of death and disability globally (Feigin et al., 2021) and in Switzerland ("Herz- und Kreislauf-Erkrankungen", 2024). In 2022, 21'797 individuals in Switzerland experienced a stroke ("Herz- und Kreislauf-Erkrankungen", 2024). With a lifetime prevalence of 3.5% in the Swiss population, it is estimated that over 300'000 people will experience a stroke in their lifetime ("Diseases", 2024). About one-third of the individuals experiencing a stroke die, leaving one half of stroke survivors permanently disabled ("WHO EMRO — Stroke, Cerebrovascular accident — Health topics", 2024).

UL dysfunction is the most common disabling deficit following a stroke (Faria-Fortini et al., 2011). These impairments include muscle weakness, spasticity, loss of fine motor control, and sensory deficits, which collectively limit activities such as grasping, lifting, and reaching. These challenges are particularly pronounced in stroke patients suffering from hemiplegia, a full or partial paralysis of the muscles of the lower face, arm, and leg on one side of the body. For instance, muscle weakness in the paretic UL is strongly correlated with a reduced capacity to perform activities of daily living (ADL) (Harris and Eng, 2007).

Spasticity, another common impairment, develops in nearly half of stroke survivors within the first year. This condition can further restrict joint movement and exacerbate pain, significantly hindering functional recovery (Opheim et al., 2014). Additionally, sensory impairments such as numbness, tingling, or reduced proprioception contribute to difficulties in grip control and precision tasks (Carlsson et al., 2018). These deficits significantly impact survivors' independence and quality of life, with only 5-20% of stroke survivors regaining full functional use of their affected

UL (Kwakkel et al., 2003).

1.2 Multiple Sclerosis

Multiple sclerosis (MS) is a chronic autoimmune disease characterized by inflammation and neurodegeneration within the central nervous system. This condition leads to demyelination and axonal loss, resulting in a variety of neurological deficits, including UL impairments in over 60% of MS patients. They frequently experience reduced strength and impaired dexterity in the UL. These limitations affect their ability to perform fine motor tasks such as using utensils, typing, or writing, which are crucial for daily activities and independence (Marrie et al., 2017). Furthermore, UL movements are often less coordinated and less smooth in MS patients. Altered muscle activation patterns and irregular trajectories are common, especially during tasks requiring precision, such as reaching or grasping objects (Pellegrino et al., 2018). Trunk control deficits, a common issue in MS, further exacerbate UL dysfunction. Impaired postural stability reduces voluntary control of the arms, making tasks such as lifting or stabilizing objects even more challenging (Cetisli Korkmaz et al., 2018).

MS is associated with microstructural changes in white matter that correlate with motor impairments in UL. These changes suggest the potential for neuroplasticity and highlight the importance of interventions aimed at motor rehabilitation (Bonzano et al., 2014). Even in the early stages of MS, bilateral UL dysfunction is often observed (Bertoni et al., 2015). UL impairments significantly reduce quality of life for MS patients. Loss of independence, increased reliance on assistive devices, and higher rates of depression are commonly reported (Alonso et al., 2021). Rehabilitation programs focusing on task-specific exercises and motor coordination have shown promise in improving UL function and delaying progression (Lamers et al., 2016).

1.3 Brachial Plexus Injury

Brachial plexus injury (BPI) is a severe peripheral nerve injury that significantly impairs UL function, leading to motor, sensory, and quality of life challenges. This injury can result from trauma, such as high-velocity accidents, or medical conditions, such as tumors or compression syndromes (Park et al., 2017). BPI often causes partial or complete paralysis of the shoulder, arm, and hand, depending on the extent of nerve damage. Injuries affecting the C5–C6 roots typically impair shoulder abduction and elbow flexion, while lower plexus injuries (C8–T1) can result in weak grip and finger movements, with some cases leading to total arm paralysis (Huang et al., 2021).

Patients frequently experience diminished or absent sensation in affected areas, with injury severity dictating the extent of sensory loss. For example, damage to the lower roots (C8–T1) affects tactile sensation on the ulnar side of the hand, while injuries to upper roots (C5–C6) impact the shoulder and lateral arm (Bertelli et al., 2011). Sensory impairments may also manifest in the contralateral limb due to neural plasticity following unilateral injury (Ramalho

et al., 2019). Neuropathic pain is a common and often debilitating symptom, especially in cases involving nerve root avulsion. This pain can persist despite conservative or surgical treatments, severely affecting patients' well-being (Bertelli et al., 2011).

BPI also significantly diminishes quality of life, particularly in adults, where injuries often preclude employment and limit social participation. Patients frequently rely on assistive devices or surgical interventions, such as nerve grafts or tendon transfers, which aim to restore partial function (Holdenried et al., 2013). Rehabilitation, including physiotherapy and nerve transfer surgeries, has shown promise in improving outcomes, with many patients regaining functional independence over time (Popova, 2022).

1.4 Laing Distal Myopathy

Laing distal myopathy (LDM) is a rare, autosomal dominant myopathy caused by mutations in the MYH7 gene, characterized by early-onset often before five years old and results in weakness predominantly in the distal muscles, including those of the UL. Affected individuals typically exhibit progressive weakness in the finger extensors alongside neck flexor and ankle dorsiflexion impairments, with proximal muscle involvement becoming apparent as the disease advances (Lamont and Laing, 2021). Muscle biopsies in affected individuals frequently show variability in fiber size and structural abnormalities like rimmed vacuoles and mitochondrial changes, indicative of underlying myofibrillar disruption (Tasca et al., 2012). Management currently focuses on symptomatic treatment, including physiotherapy to maintain mobility and prevent contractures, but no clinical practice guidelines for LDM have been published (Lamont and Laing, 2021).

1.5 Poliomyelitis

Poliomyelitis, often referred to as polio, is a highly contagious viral disease caused by poliovirus. It mostly spreads through person-to-person contact via the fecal-oral route and can lead to severe neurological complications. While many infected individuals remain asymptomatic, the virus can infect the central nervous system, resulting in muscle weakness or paralysis. The disease mostly affects children under five years old. However, polio is rare due to effective vaccines (Walter and Malani, 2022).

The phenomenon of post-polio syndrome also contributes to the progressive muscular atrophy and weakness in previously unaffected or partially recovered muscles, with the UL commonly involved. These impairments include symptoms such as muscle weakness, often associated with joint pain and mental and physical fatigue (Walter and Malani, 2022), showing a decline in ADL in their tested limb (Allen et al., 2004). Polio survivors are at risk for conditions such as median and ulnar nerve entrapments, due to repetitive hand movements or weight-bearing activities associated with mobility aids dependency like wheelchairs and crutches (Tsai et al., 2009). This again can

lead to pain and decreased functional independence, thereby leading to a diminished quality of life.

2 The Myoshirt

The Myoshirt consists of two main components: the Tendon Driver Unit (TDU) and the textile interface. The TDU serves as the core of the system, housing essential actuation components including a motor, battery, and microcontroller. This unit is mounted on the back of the user's torso using a padded hip belt, ensuring portability and independence from external power sources. Weighing just 1.8 kilograms, the TDU enables full mobility while generating the forces necessary to partially support arm movements. This force is transmitted through a tendon system integrated into the textile interface. The textile interface includes a modular lightweight load-carrying equipment vest and an arm cuff. The tendon is routed through a guide positioned at the shoulder and attached to the cuff on the upper arm. This configuration provides structural stability and ensures that the assistance is effectively applied to the arm. Additionally, the system incorporates an Inertial Measurement Unit (IMU), which is worn on the upper arm and continuously monitors arm elevation. The IMU transmits real-time data to the microcontroller within the TDU, which calculates the torque required to offset a percentage of the gravity torque of the arm based on a compensation model derived from anthropometric data. The motor then supplies this torque, transforming it into a linear force that is transmitted through the cable to the cuff. By compensating for gravity, the Myoshirt aims to reduce the muscular effort required for lifting or holding the arm, especially during shoulder flexion and abduction tasks, thereby easing movement.

The level of gravity compensation can be adjusted via software to meet individual users' needs. Users can specify the percentage of gravity to be compensated, ranging from 0% to 100%. However, practical experience has shown that support levels exceeding 50% can lead to discomfort due to the reaction forces exerted on the user's shoulder and back. As a result, support levels are typically optimized between 30% and 50% to balance assistance with user comfort. The design of the Myoshirt and functionality aim to find usages in both therapeutic and daily living contexts. In rehabilitation settings, the device could support patients during physiotherapy or occupational therapy sessions, aiming to facilitate motor learning and functional recovery, specifically when performing repetitive exercises or training ADL tasks. By reducing the physical burden of movement, it allows them to perform their exercises with better form and higher repetitions. Beyond therapy, the Myoshirt should serve as an assistive device for individuals with chronic impairments. By providing continuous support during ADL, it aims to enable the user to perform tasks such as eating, drinking, or reaching for objects with greater ease. Its portable and lightweight design allows for use at home or in community environments, promoting independence and improving overall quality of life.

While the current design of the Myoshirt supports only one arm at a time, a newer device aims to expand its functionality to include support for both arms. This is particularly relevant for users who require bilateral assistance.

The Myoshirt represents a step forward in the development of wearable exosuits. This device not only tries to address the immediate needs of individuals with UL impairments, but also holds the potential to reshape how assistive technologies are integrated into both clinical and everyday settings. This prototype also aims to give general insights into the impact of an UL exosuit on various tasks.

3 Participant 004 Results

The results for this participant are reported separately to ensure clarity and transparency, as these measurements were conducted differently and using a goniometer and visual assessments.

3.1 Reachable Work Area

The reachable work area task was performed on paper, with the participant using a pen to draw circles under both conditions. A visual comparison of the drawn circles showed no noticeable difference in size between the two conditions.

3.2 Shoulder Abduction and Flexion

Shoulder abduction increased from 55 without the Myoshirt to 71 with the Myoshirt, representing a percentage increase of 29.09% compared to the baseline without the device. Shoulder flexion also increased, rising from 70 without the Myoshirt to 75 with it, resulting in a percentage change from the baseline of 7.14%.

3.3 Elbow Flexion and Extension

Elbow ROM was assessed during maximum shoulder flexion. Without the Myoshirt, the participant demonstrated a maximum elbow flexion angle of 122 and an elbow extension angle of 10 during 70 shoulder flexion. With the Myoshirt, during 75 shoulder flexion, themaximum elbow flexion angle decreased to 111, while the extension angle slightly improved to 6. This resulted in a percentage change of -6.25% in total elbow ROM when supported by the Myoshirt compared to the baseline condition without the device.

4 Qualitative Feedback

Participants provided a wide range of feedback on the usability, comfort, and functionality of the Myoshirt. Participant 001 found the Myoshirt too uncomfortable to continue using, citing issues with the back-mounted box and suggesting a front-mounted alternative, while acknowledging that it might not be compatible with the rope system. Participant 002 experienced discomfort due to the shirt's heavy pressure on the shoulder, with visible pulling from the cord, possibly due to an improper fit for the participant's weight or height.

Participant 003 expressed a need for elbow support, feeling that the upper arm assistance was less beneficial without corresponding support for the lower arm. Participant 004 praised the shirt's design as appealing and suitable for "badass" activities and suggested a locking system for static tasks such as toothbrushing. They also noted minor discomfort from tension in the cord and slippage of the cuff but found the box comfortable. Participant 005 described the shirt as feeling like a heavy backpack and suggested wrist or forearm support to assist with fine motor tasks like gripping.

Participant 006 experienced backward pulling during the drinking task, which hindered performance. Participant 007 reported that the cable of the shirt interfered with task execution, including pulling at incorrect times during the toothbrush task, restricting forward arm motion in the cup task, and pulling the shoulder backward in the bag task. General feedback from them highlighted that the shirt sometimes hindered movements instead of supporting them, and the participant felt compression on the shoulder.

Participant 008 reported reduced shoulder pain due to subluxation while wearing the Myoshirt, which improved stabilization and allowed for better control and focus during tasks. Participant 009 observed improved movement in certain ADL tasks, such as reduced elbow rotation in the drawer task, but experienced fit issues due to slim body proportions, leading to discomfort from pulling in the shoulder and neck area. They expressed optimism about the shirt's future development and potential utility once refined.

The qualitative feedback aligns with the questionnaires and underscores both the potential and limitations of the Myoshirt, with participants highlighting key issues related to comfort, functionality, and suitability for different levels of impairment. Comfort emerged as a major concern, particularly due to the TDU placement, cable tension, and shirt tightness, suggesting a need for more ergonomic designs and adjustable sizing to accommodate diverse body types.

The feedback revealed that participants with mild impairments often found the Myoshirt more hindering than helpful, as its supportive forces could interfere with natural movements. In contrast, participants with more severe impairments reported benefits such as improved stabilization and reduced pain from shoulder subluxation, emphasizing the importance of targeting the Myoshirt to individuals with significant functional limitations.

Functionality issues included challenges with control algorithms, hardware and cable mechanics, which at times

applied forces in counterproductive ways, limiting the utility of the Myoshirt for dynamic tasks. Additionally, the lack of support for the lower arm was identified as a limitation, particularly for participants with fine motor impairments.

5 Therapist Ratings and Comments

5.1 P001

Therapist Name: J.M.

Date Rated: 2024-10-30

ADL Tasks w/o Shirt	Recording No.	Therapist Rating	Comments
1: Opening Drawer	0028-0030	2	slips fingers on second attempt, left pinky contracted, uses first two digits primarily
2: Retrieving Towel	0031-0033	2	grasps at towel twice before lifting on first attempt, needs to adjust grasp on third attempt
3: Toothbrush	0034-0036	1	normal bilateral control and speed
4: Cup	0037-0039	1	able to hold 5 sec, left pinky finger contracted
5: Cookie	0040-0042	1	able to hold 5 sec
6: Key	0043-0045	1	able to place in first attempt
7: Phone	0046-0048	1	normal
8: Handbag	0049-0052	1	normal, left pinky contracted

ADL Tasks w/ Shirt	Recording No.	Therapist Rating	Comments
1: Opening Drawer	-		n/a no recordings did not wear shirt
2: Retrieving Towel	-		n/a no recordings did not wear shirt
3: Toothbrush	-		n/a no recordings did not wear shirt
4: Cup	-		n/a no recordings did not wear shirt
5: Cookie	-		n/a no recordings did not wear shirt
6: Key	-		n/a no recordings did not wear shirt
7: Phone	-		n/a no recordings did not wear shirt
8: Handbag	-		n/a no recordings did not wear shirt

5.2 P002

Therapist Name: J.M.

Date Rated: 2024-10-30

ADL Tasks w/o Shirt	Recording No.	Therapist Rating	Comments
1: Opening Drawer	0029-0031	1	fingers slip first attempt
2: Retrieving Towel	0033-0035	1	some increased trunk sway
3: Toothbrush	0036-0039	1	normal
4: Cup	0040-0042	1	4 sec each time but probably just too fast
5: Cookie	0043-0045	1	4 sec each time but probably just too fast
6: Key	0046-0048	1	slides key to edge to grasp, last attempt pinches to pick up
7: Phone	004-0051	1	normal
8: Handbag	0052-0054	1	normal, slow

ADL Tasks w/ Shirt	Recording No.	Therapist Rating	Comments
1: Opening Drawer	0082-0084	1	arm jerks back on first trial when returning to resting position, the cable looks like it is crossing and pressing on front of arm
2: Retrieving Towel	0085-0087	1	normal, no trunk sway
3: Toothbrush	0088-0090	1	right arm actually lifts higher
4: Cup	0091-0093	1	smooth, doesn't hold 5 sec
5: Cookie	0094-0096	1	doesn't hold 5 sec
6: Key	0097-0099	1	
7: Phone	0100-0102	1	
8: Handbag	0103-0105	1	

5.3 P003

Therapist Name: J.M.

Date Rated: 2024-10-30

ADL Tasks w/o Shirt	Recording No.	Therapist Rating	Comments
1: Opening Drawer	0017-0019	3	cogging motion on intention, poor ability to lift left hand to table
2: Retrieving Towel	0020-0022	3	slow and difficut lifting out of drwaer, multiple adjustments of grip on towel; trunk sway with max shoulder flexion
3: Toothbrush	0023-0024	2	drops toothpaste first attempt; slow
4: Cup	0025-0026	4	multi-step raising arm to get to mouth, tremor of hand, diskinetic path to mouth, unable to hold for more than a couple seconds
5: Cookie	0027-0028	2	slow, able to bring to mouth and hold a few seconds
6: Key	0029-0030	2	able to pinch key, dysmetria to place key but able to do it, cogging
7: Phone	0031-0032	4	difficulty holding phone and doesn't place to ear
8: Handbag	0033-0034	5	uses momentum to swing bag onto table but unable to lift to top of drawer

ADL Tasks w/ Shirt	Recording No.	Therapist Rating	Comments
1: Opening Drawer	0047-0049	1	smoother and improved flexion rom
2: Retrieving Towel	0050-0051	1	can bring towel out in one motion, initial lift is smooth and high
3: Toothbrush	0052-0053	2	no tremor
4: Cup	0054-0055	4	able to bring to mouth but not able to hold, slow, cogging on second attempt can bring to mouth but 1 sec
5: Cookie	0056-0057	2	holds at mouth 2 seconds
6: Key	0058-0060	4	uses right hand to assisting to get into key slot first attempt; second attempt pushes slot over and jamming it in, third attempt did it great
7: Phone	0061-0062	4	lifted to mouth again, puts on cheek second attempt
8: Handbag	0063-0064	5	uses momentum to lift onto the table

5.4 P004

Therapist Name: J.M.

Date Rated: 2024-10-30

ADL Tasks w/o Shirt	Recording No.	Therapist Rating	Comments
1: Opening Drawer	0002	2	elbow flexion contracture, only did task once
2: Retrieving Towel	0003	2	"
3: Toothbrush	0004	1	"
4: Cup	0005	2	" only held cup up (mouth out of view) for a little over a second
5: Cookie	0006	2	brings up but still in frame and does not hold at mouth
6: Key	0007	1	elbow flexion contracture, only did task once
7: Phone	0008	3	out of phrame but doesn't hold for 5 seconds, no shoulder abduction, extreme wrist extension
8: Handbag	0009	5	placed on the table, not clear if that was instructions or if patient has weakness

ADL Tasks w/ Shirt	Recording No.	Therapist Rating	Comments
1: Opening Drawer	0011		not recorded
2: Retrieving Towel	0012	1	did faster
3: Toothbrush	0014	1	normal
4: Cup	0013	1	did after retieving towel
5: Cookie	0015	1	
6: Key	0016	1	
7: Phone	0017	1	
8: Handbag	0018	5	brings up onto table

5.5 P005

Therapist Name: J.M.

Date Rated: 2024-10-30

ADL Tasks w/o Shirt	Recording No.	Therapist Rating	Comments
1: Opening Drawer	0027-0029	3	tremor on first reach with supinating hand, only has control of first finger; trunk lean instead of elbow extension to reach drawer
2: Retrieving Towel	0030-0032	3	trunk lean to get towel out, unable to maintain strong grasp, uses momentum to take towel out
3: Toothbrush	0033-0035	4	needs to reposition toothbrush in hand, drops on second attempt, uses trunk to bring arms together, drops brush on third attempt
4: Cup	0036-0038	4	uses trunk to bring arm to chest, hand tremor and significant difficulty to hold cup, unable to raise to mouth, fingers contracted around handle after grasp, closer to mouth second attempt and able to hold several seconds, third attempt looks most normal but significant trunk lean.
5: Cookie	0039-0041	4	cannot grasp cookie well, falling out of hand, brings face to hand, unable to hold, able to at last trial with pinch between fingers
6: Key	0042-0044	4	difficulty pinching key and unable to place in slot first attempt, uses right hand to place key in left then uses left hand assist to help right place the key, repeats on third and able to perform spoothly with right hand assist
7: Phone	0045-0047	4	uses trunk and right to assist phone in and out of left; able to bring to ear with head flexed compensation; on last attempt able to do completely with left hand but phone gets stuck between fingers and has to use side of face to prevent phone from slipping
8: Handbag	0048-0050	4	two steps to put on drwarer first needs to lift to table then uses right hand to help reposition, only lifts onto table for last two trials.

ADL Tasks w/ Shirt	Recording No.	Therapist Rating	Comments
1: Opening Drawer	0067-0069	2	faster
2: Retrieving Towel	0070-0072	2	improved lift of arm and less trunk sway to reach towel, able to grasp and hold towel
3: Toothbrush	0073-0075	3	still needs to put toothbrush in hand, less trunk motion
4: Cup	0076-0078	2	able to bring to mouth with two assist for r hand to position cup, second and third attempt able to bring to mouth with posterior trunk lean
5: Cookie	0079-0081	3	uses right hand to place in left, then trunk extension to bring hand to mouth
6: Key	0082-0084	4	able to get key in on first try without use of right hand, improvied shoulder abduction, key falls out second and third attempts, improved time holding arm in abduction and elevation to attempt to get it into the slot
7: Phone	0085-0087	4	uses right hand to place in left, then trunk extension to bring hand to cheek and unable to hold
8: Handbag	0088-0090	4	able to bring onto table in one motion, attempts to try to put on top of drawer but uses right arm to complete; difficulty with right hand grabbig straps of bag

5.6 P006

Therapist Name: J.M.

Date Rated: 2024-10-30

ADL Tasks w/o Shirt	Recording No.	Therapist Rating	Comments
1: Opening Drawer	0027-0029	1	very slight challenge with supinating hand
2: Retrieving Towel	0030-0032	1	slow
3: Toothbrush	0033-0035	1	normal
4: Cup	0036-0038	1	3 sec first attempt, 5 sec second attempt, 4 sec
5: Cookie	0039-0041	1	didnt hold for 5 sec on any trials but had smooth motion
6: Key	0032-0044	1	twists key to put in on second attempt
7: Phone	0045-0047	1	slow, didn't hold 5 sec
8: Handbag	0048-0050	2	got fingers stuck around handlle, needed to rest on table first, second attempt can do in one go, third attempt needs to rest on table

ADL Tasks w/ Shirt	Recording No.	Therapist Rating	Comments
1: Opening Drawer	0067-0069	1	maybe slightly faster
2: Retrieving Towel	0070-0072	1	normal
3: Toothbrush	0073-0075	1	normal, slow
4: Cup	0076-0078	1	doesnt hold for 5 sec. seems like brings head to cup more this time
5: Cookie	0079-0081	1	doesnt hold for 5 sec
6: Key	0082-0084	1	
7: Phone	0085-0087	1	doesn't hold 5 ec, faster
8: Handbag	0088-0090	2	brings onto table brielfly then able to lift up onto drawer

5.7 P007

Therapist Name: J.M.

Date Rated: 2024-10-30

ADL Tasks w/o Shirt	Recording No.	Therapist Rating	Comments
1: Opening Drawer	0030-0032	3	no ability to elbow flex, finger crawls hand across table to reach drawer, difficulty returning to pronation
2: Retrieving Towel	0033-0035	3	extraneous shoulder movement, also unable to lifth right hand off table and crawls hand
3: Toothbrush	0036-0038	2	able to perform with hands close to body, needed to adjust paste in hand
4: Cup	0039-0041	3	able to bring to mouth with elbow flexion close to body and hold it, slight trunk lean, didn't bring completely to mouth
5: Cookie	0042-0044	2	looks smooth and able to hold, second trial slaps hand onto cookie
6: Key	0045-0047	3	severe difficulty bringing hand up to keyhole, able to with r hand assist, brings arm across midline and flexes elbow and supinates wrist due to lack of shoulder flexion strength but able to complete quickly and smoothly
7: Phone	0048-0050	2	shoulder elevation to assist in bringing the phone to ear
8: Handbag	0051-0053	5	able to bicep curl to the table but unable to lift on top of drawer

ADL Tasks w/ Shirt	Recording No.	Therapist Rating	Comments
1: Opening Drawer	0069-0071	3	camera blurry, maybe faster, still crawls hand
2: Retrieving Towel	0072-0074	3	still needs to walk hands to drawer, but initial lift to get hand into drawer muich higher, and able to do smoother and quicker
3: Toothbrush	0075-0077	2	brings hands close to body to apply paste, uses bottle against left hand to slide right down tubing
4: Cup	0078-0080	2	able to bring to closer to mouth with elbow flexion close to body and hold it, less trunk lean
5: Cookie	0081-0083	2	keeps arm close to body
6: Key	0084-0087	2	uses both hands
7: Phone	0088-0090	2	less shoulder elevation
8: Handbag	0091-0093	5	able to bring to table

5.8 P008

Therapist Name: J.M.

Date Rated: 2024-02-26

ADL Tasks w/o Shirt	Recording No.	Therapist Rating	Comments
1: Opening Drawer		3	unable to lift forearm, wrist, hand off table, uses creep technique with first two fingers and thumb, pushes back in with thumb and trunk rotation and flexion compensation
2: Retrieving Towel		3	lateral trunk flexion compensation to bring hand up and in to drawer, compensation with inertia to bring arm back onto table, able to open and close hand with increased wrist flexion
3: Toothbrush		4	able to lateral lean to lift hand <2inches off table, difficulty grasping brush into hand, achieves by third trial
4: Cup		5	lateral trunk lean compensation, unable to complete elbow flexion without unaffected hand assist, able to maintain grasp on cup handle with affected fingers but requires assist from other hand for initial positioning
5: Cookie		5	same as cup, cannot complete without unaffected hand assist, second trial incrased shoulder elevation and on third trial biceps activity observed on initial elbow flexion
6: Key		5	able to grasp key but requires uninvolved limb support to stabilize wrist and elevate hand to key hole height, needs unaffected hand assist for initial key positioning except last trial
7: Phone		5	able to grasp phone with effort and trunk lean, unable to complete elbow flexion to ear without unaffected limb assist
8: Handbag		5	uses momentum of arm swing and trunk lean with elbow in extension to brung bag onto table, unable to bring to drawer height

ADL Tasks w/ Shirt	Recording No.	Therapist Rating	Comments
1: Opening Drawer		2	same technique as w/o, increased elbow heiight pushing drawer back in with less trunk compensation
2: Retrieving Towel		2	slightly less trunk lean to bring arm in/out of drawer, less use of momentum, increased activation of shoulder elevators and flexors, less overall displacement of hand and lower arm
3: Toothbrush		3	improved ability to grip brush, lateral compensation present, increased consistency lifting hand off table
4: Cup		4	improved elebow flexion noted, continues to require unaffected hand support but less; can't rule out an order effect for increased elbow flexor recruitment, but shoulder appears more stable
5: Cookie		5	improved elbow flexion and shoulder stability for initial elbow flexion motion, continues to require unaffected hand compensation, can't rule out order effect
6: Key		5	still using unaffected hand to assist to lift, appears to have myoshirt support greatest at end range shoulder flexion at end of movement
7: Phone		5	uses unaffected arm to complete elbow ROM entirely, trunk lean persists to lift hand to grasp phone initially
8: Handbag		5	continues to use momentum, only able to bring bag onto table

5.9 P009

Therapist Name: J.M.

Date Rated: 2024-02-26

ADL Tasks w/o Shirt	Recording No.	Therapist Rating	Comments
1: Opening Drawer		3	able to lift wrist, difficulty extending shoulder and compensates with biceps activation/elbow flexion, drawer not fully open, able to pronate and supinate forearm; one rep
2: Retrieving Towel		3	uses momentum and elbow flexion to bring hand into drawer, uses wrist supination and finger creep to bring towel out, no eccentric control to lower hand to table, slight lateral trunk lean compensation, one rep
3: Toothbrush		2	able to internally rotate and adduct shoulder for hand to grap brush, slight trunk rotation compensation, one rep
4: Cup		5	able to bring hand to cup with shoulder elevation compensation, unable to bring cup to mouth remaining 50% of elbow ROM, uses other hand to hold static position; one rep
5: Cookie		2	neck flexion to bring mouth to cookie, shoulder elevation to reach for cookie in beginning and maintain static position at end, one rep
6: Key		5	biceps activation and trunk rotation, able to grasp key and flex elbow to 90 degrees but unable to lift elbow off of table against gravity; one rep
7: Phone		2	able to complete with elbow supported on the table; one rep
8: Handbag		5	able to lift bag to table height with trunk extension and max elbow flexion, no shoulder flexion noted

ADL Tasks w/ Shirt	Recording No.	Therapist Rating	Comments
1: Opening Drawer		2	elbow lifts off of table, able to open drawer greater distance, increased shoulder abduction and appropriate biceps activation, elevated shoulder with effort still present; one rep
2: Retrieving Towel		2	elebow elevated throughout , able to reduce shoulder elevation compensation, wrist remains level and no loss of eccentric control to bring arm back to table; one rep
3: Toothbrush		1	able to lift hand and elbow off table to apply paste, no trunk compensation; one rep
4: Cup		2	makes it 3/4 of the way to mouth, able to hold for a solid 9 sec and able to control eccentrically to lower hand back to table; one rep
5: Cookie		1	no neck flexion compensation, maintains shoulder elevation but improved flow of movement and ability to hold for time; one rep
6: Key		3	able to lift elbow to level of key hole and extend elbow to slot when using unaffected hand to stabilize under the affecgted elbow. able to complete task after prolonged attempt; one rep
7: Phone		1	maintains shoulder abduction , smooth movement, elbow stays elevated; one rep
8: Handbag		5	improved shouler abduction, still can only bring to table; one rep

References

Allen, G. M., Middleton, J., Katrak, P. H., Lord, S. R., & Gandevia, S. C. (2004). Prediction of voluntary activation, strength, and endurance of elbow flexors in postpolio patients. *Muscle Nerve*, *30*(2), 172–181. https://doi.org/10.1002/MUS.20094

Alonso, R. N. Et al. (2021). Upper limb dexterity in patients with multiple sclerosis. *Int J MS Care*, *23*(2), 79–84. https://doi.org/10.7224/1537-2073.2019-083

Bertelli, J. A., Ghizoni, M. F., & Loure Iro Chaves, D. P. (2011). Sensory disturbances and pain complaints after brachial plexus root injury: A prospective study involving 150 adult patients. *Microsurgery*, *31*(2), 93–97. https://doi.org/10.1002/MICR.20832

Bertoni, R., Lamers, I., Chen, C. C., Feys, P., & Cattaneo, D. (2015). Unilateral and bilateral upper limb dysfunction at body functions, activity and participation levels in people with multiple sclerosis. *Multiple Sclerosis Journal*, *21*(12), 1566–1574. https://doi.org/10.1177/1352458514567553

Bonzano, L. Et al. (2014). Upper limb motor rehabilitation impacts white matter microstructure in multiple sclerosis. *Neuroimage*, *90*, 107–116. https://doi.org/10.1016/J.NEUROIMAGE.2013.12.025

Carlsson, H., Gard, G., & Brogårdh, C. (2018). Upper-limb sensory impairments after stroke: Self-reported experiences of daily life and rehabilitation. *J Rehabil Med*, *50*(1), 45–51. https://doi.org/10.2340/16501977-2282

Cetisli Korkmaz, N., Can Akman, T., Kilavuz Oren, G., & Bir, L. S. (2018). Trunk control: The essence for upper limb functionality in patients with multiple sclerosis. *Mult Scler Relat Disord*, *24*, 101–106. https://doi.org/10.1016/j.msard.2018.06.013

Diseases [Accessed: Dec. 09, 2024]. (2024). https://www.bfs.admin.ch/bfs/en/home/statistics/health/state-health/diseases.html

Faria-Fortini, I., Michaelsen, S. M., Cassiano, J. G., & Teixeira-Salmela, L. F. (2011). Upper extremity function in stroke subjects: Relationships between the international classification of functioning, disability, and health domains. *Journal of Hand Therapy*, *24*(3), 257–265. https://doi.org/10.1016/j.jht.2011.01.002

Feigin, V. L. Et al. (2021). Global, regional, and national burden of stroke and its risk factors, 1990–2019: A systematic analysis for the global burden of disease study 2019. *Lancet Neurology*, *20*(10), 795–820. https://doi.org/10.1016/S1474-4422(21)00252-0

Harris, J. E., & Eng, J. J. (2007). Paretic upper-limb strength best explains arm activity in people with stroke. *Phys Ther*, *87*(1), 88–97. https://doi.org/10.2522/PTJ.20060065

Herz- und kreislauf-erkrankungen [Accessed: Dec. 09, 2024]. (2024). https://www.bfs.admin.ch/bfs/de/home/statistiken/gesundheit/gesundheitszustand/krankheiten/herz-kreislauf-erkrankungen.html

Holdenried, M., Schenck, T. L., Akpaloo, J., Müller-Felber, W., Holzbach, T., & Giunta, R. E. (2013). Quality of life after brachial plexus lesions in adults. *Handchirurgie, Mikrochirurgie, plastische Chirurgie*, *45*(4), 229–234. https://doi.org/10.1055/S-0033-1353161

Huang, H. Et al. (2021). Therapeutic strategies for brachial plexus injury. *Folia Neuropathol*, *59*(4), 393–402. https://doi.org/10.5114/FN.2021.111996

Kwakkel, G., Kollen, B. J., Van der Grond, J. V., & Prevo, A. J. H. (2003). Probability of regaining dexterity in the flaccid upper limb: Impact of severity of paresis and time since onset in acute stroke. *Stroke*, *34*(9), 2181–2186. https://doi.org/10.1161/01.STR.0000087172.16305.CD

Lamers, I. Et al. (2016). Upper limb rehabilitation in people with multiple sclerosis. *Neurorehabil Neural Repair*, *30*(8), 773–793. https://doi.org/10.1177/1545968315624785

Lamont, P., & Laing, N. G. (2021). *Laing distal myopathy* [Accessed: Dec. 09, 2024]. https://www.ncbi.nlm.nih.gov/books/NBK1433/

Marrie, R. A., Cutter, G. R., Tyry, T., Cofield, S. S., Fox, R., & Salter, A. (2017). Upper limb impairment is associated with use of assistive devices and unemployment in multiple sclerosis. *Mult Scler Relat Disord*, *13*, 87–92. https://doi.org/10.1016/j.msard.2017.02.013

Opheim, A., Danielsson, A., Murphy, M. A., Persson, H. C., & Sunnerhagen, K. S. (2014). Upper-limb spasticity during the first year after stroke: Stroke arm longitudinal study at the university of gothenburg. *Am J Phys Med Rehabil*, *93*(10), 884–896. https://doi.org/10.1097/PHM.0000000000000157

Park, H. R., Lee, G. S., Kim, I. S., & Chang, J.-C. (2017). Brachial plexus injury in adults. *The Nerve*, *3*(1), 1–11. https://doi.org/10.21129/NERVE.2017.3.1.1

Pellegrino, L., Coscia, M., Muller, M., Solaro, C., & Casadio, M. (2018). Evaluating upper limb impairments in multiple sclerosis by exposure to different mechanical environments. *Scientific Reports*, *8*(1), 1–14. https://doi.org/10.1038/s41598-018-20343-y

Popova, N. (2022). Complex recovery of the upper limb after nerve transfers in patients with adult brachial plexus injuries, In *Proceeding book vol.2*. https://doi.org/10.37393/ICASS2022/147

Ramalho, B. L., Rangel, M. L., Schmaedeke, A. C., Erthal, F. S., & Vargas, C. D. (2019). Unilateral brachial plexus lesion impairs bilateral touch threshold. *Front Neurol*, *10*(AUG). https://doi.org/10.3389/FNEUR.2019.00872

Tasca, G. Et al. (2012). New phenotype and pathology features in myh7-related distal myopathy. *Neuromuscular Disorders*, *22*(7), 640–647. https://doi.org/10.1016/j.nmd.2012.03.003

Tsai, H. C. Et al. (2009). Prevalence and risk factors for upper extremity entrapment neuropathies in polio survivors. *J Rehabil Med*, *41*(1), 26–31. https://doi.org/10.2340/16501977-0290

Walter, K., & Malani, P. N. (2022). What is polio? *JAMA*, *328*(16), 1652–1652. https://doi.org/10.1001/JAMA.2022.17159

Who emro — stroke, cerebrovascular accident — health topics [Accessed: Dec. 09, 2024]. (2024). https://www.emro.who.int/health-topics/stroke-cerebrovascular-accident/index.html
